# The CsFT-FD complex controls flowering by regulating *CsAP1* and *CsLFY* in saffron

**DOI:** 10.1080/21645698.2026.2651568

**Published:** 2026-04-01

**Authors:** Xiaoyuan Xi, Mengqing Feng, Jing Li, Xiaodong Qian, Jing Chen, Xiang Yu, Liqin Li

**Affiliations:** a TCM (Traditional Chinese Medicine) Key Laboratory Cultivation Base of Zhejiang Province for the Development and Clinical Transformation of Immunomodulatory Drugs, Fifth School of Clinical Medicine of Zhejiang Chinese Medical University, Huzhou, Zhejiang, China; b Affiliated Huzhou Hospital, Zhejiang University School of Medicine, Huzhou, Zhejiang, China; c School of Pharmacy, Hangzhou Normal University, Hangzhou, Zhejiang, China

**Keywords:** CsAP1, CsFT3-like-FD complex, CsLFY, flowering, saffron

## Abstract

Saffron (*Crocus sativus* L.) is a widely recognized medicinal and economic crop, valued primarily for the red stigmas that constitute its pharmaceutically active component. Flowering represents a critical agronomic trait, as it directly governs saffron yield. However, the molecular mechanisms underlying flowering regulation in saffron are not well studied. In this study, we identified a CsFT3-like-FD-AP1/LFY module involved in controlling flowering in saffron under indoor cultivation. Through transcriptomic and DAP-seq analysis, key floral identity genes *CsAPETALA1* (*CsAP1*) and *CsLEAFY* (*CsLFY*) were identified as targets of the CsFT3-like-FD complex. Direct transcriptional regulation of *CsAP1* and *CsLFY* by CsFT3-like-FD was further confirmed by dual-luciferase assays. Expression profiling revealed that *CsAP1* is predominantly expressed in saffron leaves, tepals, and stamens, whereas *CsLFY* is mainly expressed in leaves and tepals. Both proteins localize to the nucleus. Ectopic expression of *CsAP1* or *CsLFY* in *Arabidopsis* significantly accelerates flowering. Notably, *CsAP1* overexpression additionally alters floral organ architecture, indicating its dual role in promoting flowering and regulating floral organ formation. Together, these findings underscore the essential role of the CsFT3-like-FD-AP1/LFY regulatory module in saffron and offer novel insights into flowering regulation in non-model monocot plants.

## 1. Introduction

Saffron (*Crocus sativus* L.) is a valuable traditional herb and ornamental plant, prized for the unique therapeutic properties and commercial significance of its stigmas, which are widely used as a spice and colorant.^1–3^ Flowering is the key determinant of saffron yield and is regulated by both genetic and environmental factors.^4^ In indoor cultivation systems, saffron responds to environmental cues such as light and temperature.^5,6^ Notably, floral induction in saffron exhibits strong temperature dependence: high temperatures promote flowering by inducing *CsatFT3* expression and its interaction with CsatFD2 to form a florigen activation complex, while low temperatures maintain vegetative growth by upregulating *CsatSVP2* and *CsatTFL1-3/CEN1*, which respectively suppress *CsatFT3* transcription and compete with it for CsatFD2 binding.^5^

The FT-FD module represents a conserved regulatory complex in angiosperms that integrates endogenous and environmental signals to activate floral identity genes such as *AP1*, *LFY*, *CAL*, and *FUL*, thereby promoting flowering.^7–12^ *AP1*, a MIKC-type MADS-box transcription factor,^13^ acts as a class A gene in the ABCDE model.^14^ It plays a key role in coordinating the development of floral primordia by controlling genes involved in organ growth and patterning.^15^ The *ap1* mutant in *Arabidopsis* exhibits a partial transformation of flowers into inflorescence shoots, accompanied by changes in sepal and petal identity.^16^ The ectopic expression of *GmAP1* from soybean in tobacco results in early flowering and alterations of floral organs.^17,18^ Overexpression of the *OfAP1-a* gene from *Osmanthus fragrans* in *Arabidopsis* led to earlier flowering, accompanied by abnormal development of inflorescences, flowers, and siliques.^19^

LEAFY (LFY) is a pioneering transcription factor essential for specifying and sustaining floral meristems^20^ and acts as an integrator to control the floral gene network.^21^ Impairment of *LFY* function causes flowers to partially revert to inflorescence shoots,^22^ whereas its ectopic expression accelerates flowering in both model and non–model plants.^23–26^ Interestingly, *LFY* can also inhibit flower formation in mutants lacking *AP1* and *CAL*, revealing an antagonistic relationship between *LFY* and *AP1/CAL* that fine–tunes floral development.^27,28^ Given their roles in regulating flowering time and modifying floral organ characteristics, we speculated that harnessing these traits could induce beneficial phenotypic alterations, ultimately improving saffron yield.

In this study, we found that *CsFT3-like* and *CsFD* promote flowering in saffron by forming a functional CsFT-FD complex. Through transcriptomic profiling and DAP-seq analysis, *CsAP1* and *CsLFY* were identified as potential targets of the CsFT3-like-FD complex, which were further confirmed by Dual-LUC assays. *CsAP1* is predominantly expressed in the leaves, tepals, and stamens of saffron, while *CsLFY* expression is primarily localized in leaves and tepals. Both proteins display nuclear localization, consistent with their roles as transcription factors. When *CsAP1* and *CsLFY* were ectopically overexpressed in *Arabidopsis thaliana*, flowering time was significantly accelerated. Specifically, overexpression of *CsAP1* resulted in developmental abnormalities in floral organs. Overall, this regulatory framework not only deepens our understanding of the mechanisms governing saffron floral transition but also identifies potential targets for precision breeding to improve the saffron yield.

## 2. Materials and Methods

### 2.1. Plant Materials

The cultivation conditions for saffron, *Arabidopsis*, and tobacco plants are as previously reported.^29^ Corms weighing 25 ± 1 g were used for study.

### 2.2. Transgenic Plants Generation

The coding sequences of *CsFD*, *CsLFY* and *CsAP1* were amplified from cDNA and cloned into the *pCAMBIA1302* vector using Gibson assembly after purification of the PCR products. The *pro35S:CsFD*, *pro35S:CsLFY* and *pro35S:CsAP1* plasmids were introduced into *Agrobacterium tumefaciens* GV3101 and transformed into Col-0 using the inflorescence dip method.^30^

### 2.3. Callus Initiation

Apical bud tissue culture was performed in vitro as reported ^31^ with some modifications to obtain callus. First, saffron corms were rinsed under running tap water for 30 min to remove surface contaminants. The corms were then surface-sterilized by immersing them in 70% (v/v) ethanol for 3 minutes, followed by treatment with 4% sodium hypochlorite for 10 minutes. The apical buds were cut into 0.5 cm^3^ tissue blocks under sterile conditions and cultured on MS basal medium supplemented with 6-benzylaminopurine (6-BA) and α-naphthaleneacetic acid (NAA). Cultures were maintained under dark conditions at 22°C for primary callus induction.

### 2.4. Agrobacterium-Mediated Transformation in Callus

To construct *pro35S:CsFT3-like-GUS* plasmid, the coding sequence of the *CsFT3-like* was inserted into the binary vector *pCAMBIA1303* digested with *Nco*I and *Spe*I. The recombinant plasmid was transformed into *Agrobacterium tumefaciens* GV3101 using electroporation. For transformation, the callus tissues were immersed in an *Agrobacterium* suspension containing 100 μM acetosyringone for 30 min. Dried the callus on sterile filter paper and subsequently cultured on co-cultivation medium for 3 days in the dark at 22°C. Washed the co-cultured callus 5 times with MS liquid medium containing 200 mg/L cefotaxime. Dried the callus on sterile filter paper and then cultured on selective MS medium containing 2 mg/L 6-BA, 0.5 mg/L NAA and 25 mg/L hygromycin. Positive transgenic calli, identified by histochemical GUS staining, were used for RNA-sequencing analysis.

### 2.5. Co-Immunoprecipitation Analysis

To test the interactions between CsFT3-like and CsFD in plant cells, total cellular proteins were extracted from *Nicotiana benthamiana* leaves that transiently expressed the combinations of *pro35S:GFP* with *pro35S:CsFD-FLAG* and the combinations of *pro35S:CsFT3-like-GFP* with *pro35S:CsFD-FLAG*. The cellular protein extraction method and the immunoprecipitation detection method were as previously reported.^32^

### 2.6. Yeast Two-Hybrid Assay

The coding sequence of *CsFT3-like* and *CsFD* were inserted into the pGBKT7 and pGADT7 vectors, respectively, and co-transformed into AH109 using the Matchmaker™ Gold Yeast Two-Hybrid System (TaKaRa) according to the manufacturer’s instructions. The positive transformants were selected on SD/-Leu/-Trp medium. The interactions were tested on the SD/-Leu/-Trp/-His/-Ade medium with an appropriate concentration of 3-amino-1,2,4-triazole (3-AT) to suppress background growth. The interactions were evaluated after incubating for 5–7 days at 30°C.

### 2.7. Dual-Luciferase Assay

For the dual-luciferase reporter assay, the putative promoter regions of *CsLFY* (−2015 to −1 bp) and *CsAP1* (−2000 to −1 bp) were amplified from genomic DNA. These promoter fragments were subsequently cloned into the *pGII_0800-LUC* vector to generate reporter constructs. In parallel, the coding sequences of *CsFT3-like* and *CsFD* were inserted into the *pFGC5941* vector to generate effector constructs. Dual-luciferase reporter assays were conducted following the established protocol.^32,33^

### 2.8. Real-Time PCR Analysis

Total RNA was isolated from leaves, tepals, stamens, and pistils collected during three characteristic stigma pigmentation stages (yellow, orange, and red) using the RNeasy® Plant Mini Kit (Qiagen, Hilden, Germany). The qPCR assays were performed as reported.^29^ The primer sequences used for Real-time PCR were listed in Table S2.

### 2.9. Subcellular Localization

Full-length coding sequences of *CsFD*, *CsAP1*, and *CsLFY* lacking termination codons were amplified using Phanta® Max Super-Fidelity DNA Polymerase (Vazyme Biotech, China) with gene-specific primers (Table S2), followed by directional cloning into the linearized *pCAMBIA1302* vector. The subcellular localization observations were performed as reported.^29^

### 2.10. Phenotypic Observation and Statistical Analysis

Morphological observations of *Arabidopsis* plants and saffron floral buds were photographed using a digital single lens reflex camera (DSLR, Canon, DS126321). Floral organs in *Arabidopsis* were photographed using a stereo microscope (SOPTOP SZN). Flowering time was defined as the days from sowing to anthesis of the first flower and the number of the rosette leaves at bolting. Mean values ± standard deviation (SD) were presented, and statistical significance of differences was assessed using a two-tailed Student’s t-test.

### 2.11. Phylogenetic Analysis

The amino acid sequences of *CsAP1* and *CsLFY* orthologs were retrieved from NCBI (https://www.ncbi.nlm.nih.gov/). Multiple sequence alignment was generated using DNAMAN 9.0. The phylogenetic trees were constructed using the neighbor-joining method with 1000 bootstrap replicates.

### 2.12. Analysis of Gene Structure and Chromosomal Localization

Detailed genomic information for the saffron triploid genome is provided by Li et al. (2025).^34^ Based on this chromosome-level assembly and annotation of *C. sativus*, we identified the exon-intron boundaries, lengths, and chromosomal locations of the *CsAP1* and *CsLFY* genes. The structures of these genes were then visualized using the web-based tool IBS 2.0 (https://www.ibs.renlab.org/#/server?ibsId=18). Finally, chromosomal distribution maps of *CsAP1* and *CsLFY* were generated with the Gene Location Visualize function in TBtools.

### 2.13. RNA-Seq and Data Analysis

Total RNA was isolated from two types of callus, non-transgenic and *pro35S:CsFT3-like*-transgenic, using TRIzol reagent. RNA-Seq libraries were prepared and sequenced on the NovaSeq 6000 platform (BGI, Wuhan, China). The clean sequencing reads were mapped to the saffron reference genome using HISAT2 (v2.0.5). DESeq2 was used to identify Differentially Expressed Genes (DEGs) with significance criteria of *p* < 0.05 and |log_2_ fold change| ≥1.0. Functional enrichment analysis of DEGs was performed using TBtools, where Gene Ontology (GO) terms with an FDR < 0.05 were deemed significantly enriched.

### 2.14. DAP-Seq and Data Analysis

DAP-seq assays were performed by Bluescape Hebei Biotech Co., Ltd. (Baoding, China), following a previously described method.^35,36^ The Halo Tag-CsFD fusion protein was in vitro expressed using the TNT SP6 Coupled Wheat Germ Extract System (Promega) and purified with Magne Halo tag beads (Promega). The beads without the addition of CsFD protein were used as input libraries. The data were analyzed as reported.^35^

## 3. Results

### 3.1. The CsFT3-like and CsFD Proteins Form a Complex to Regulate Flowering Time

**Figure 1.**
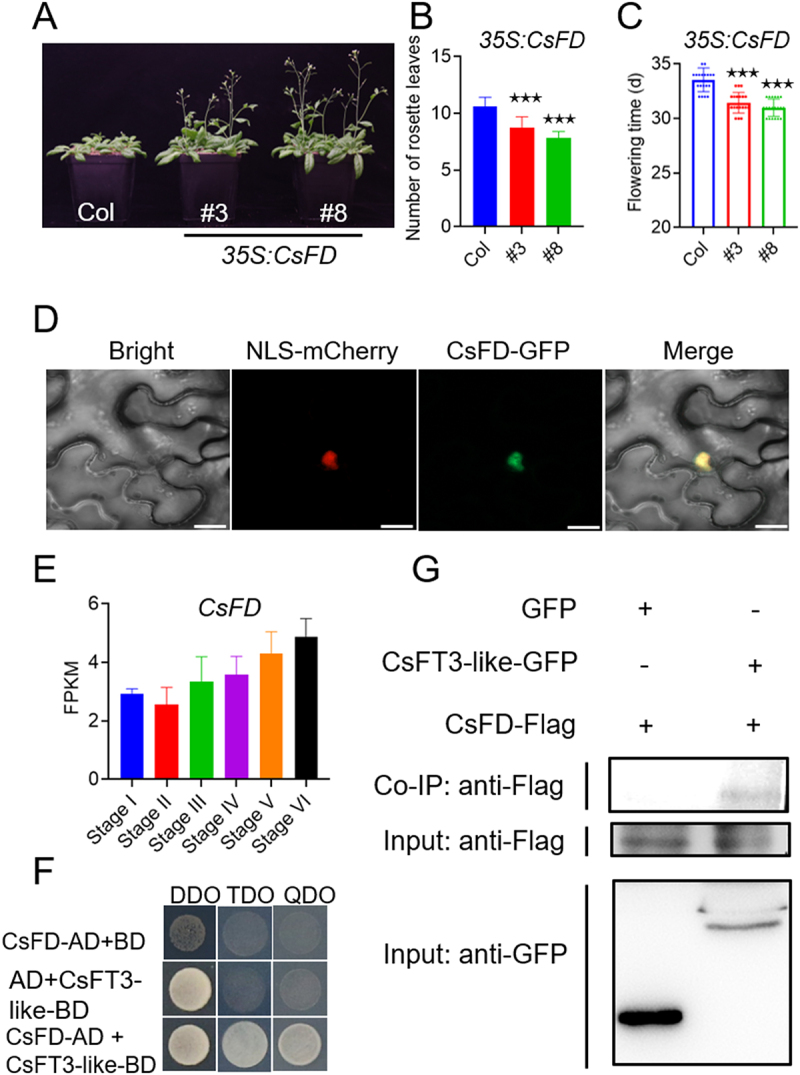
CsFD facilitates flowering and forms a complex with CsFT3-like protein. (A) Transgenic lines overexpressing *CsFD* driven by 35S promoter flowered earlier than wild-type (WT) plants under long-day (LD, 16 h : 8 h, light : dark) conditions. (B) The number of rosette leaves of *35S:CsFD* transgenic lines and WT were counted at the onset of bolting. (C) Days to flowering of *35S:CsFD* transgenic lines and WT. The data are presented as means ± standard deviation (SD). (D) The subcellular localization of CsFD protein. Scale bars represent 20 μm. (E) The expression of *CsFD* in floral buds was shown at different developmental stages. The development stage of flower buds is illustrated in Figure 7A. (F) CsFT3-like interacts with CsFD, detected by Y2H assay. DDO, double dropout (SD/-Trp/-Leu) media. TDO, triple dropout (SD/-Trp/-Leu/-His) media supplemented with 50 mM 3-AT. QDO, quadruple dropout (SD/-Trp/-Leu/-His/-Ade) media supplemented with 50 mM 3-AT. (G) The CsFD protein was co-immunoprecipitated with the CsFT3-like-GFP fusion protein.

Tsaftaris et al. (2013)^37^ identified three *FLOWERING LOCUS T* (*FT*)-*like* genes: *CsatFT1-like*, *CsatFT2-like*, and *CsatFT3-like*, and found that their expression patterns differed across various organs and throughout the diurnal cycle. Subsequently, we isolated and confirmed the flowering-promoting function of the *CsFT3-like* gene,^34^ which shares an identical amino acid sequence with the previously known *CsatFT3-like* gene. Furthermore, Kalia et al. (2025)^5^ identified three FD-like genes (*CsatFD1*, *CsatFD2*, and *CsatFD3*), highlighting the crucial role of *CsatFD2* in saffron floral induction, where it may act as a central interacting hub for both activator (FT3) and repressor (TFL1-3) proteins within the Floral Activator Complex (FAC). We cloned *CsFD*, a gene encoding a 197-amino-acid protein that is distinct from known FD-like proteins while exhibiting high homology to CsatFD1. Multiple sequence alignment revealed conserved domains in the CsFD protein, such as a leucine zipper (Figure S1). To explore its potential role in floral induction, we conducted functional characterization studies on *CsFD*. Overexpression of the *CsFD* gene in wild-type *Arabidopsis* led to an early flowering phenotype (Figure 1A–C), consistent with the effects observed in *CsFT3-like* overexpression plants.^34^ Furthermore, CsFD exhibits similar tissue and subcellular localization as CsFT3-like, being expressed in saffron’s apical buds (Figure 1E), leaves, tepals, pistils, and stamens (Figure 3B), and localizing in the nucleus (Figure 1D). The key distinction lies in CsFD exhibiting exclusive nuclear localization, whereas CsFT3-like is mainly expressed in the cell membrane and nucleus.

Based on these findings, we considered whether CsFT3-like interacts with CsFD to form a functional complex that directs the activation of genes determining floral meristem identity. To validate the potential interaction between CsFT3-like and CsFD, we performed a yeast two-hybrid (Y2H) assay. Plasmids containing CsFT3-like-BD and CsFD-AD were co-transformed into yeast cells and incubated on SD/-Trp/-Leu/-Ade and SD/-Trp/-Leu/-His/-Ade media at 30°C. The results showed that yeast cells co-expressing both CsFT3-like-BD and CsFD-AD showed vigorous growth. In contrast, the negative control did not grow, unequivocally indicating an interaction between CsFT3-like and CsFD (Figure 1F).

To further confirm the interaction between CsFT3-like and CsFD, we generated two constructs: *CsFT3-like-GFP* and *CsFD-Flag*. The two constructs were co-expressed in *Nicotiana benthamiana* epidermal cells. The results demonstrated that CsFD-Flag was successfully coimmunoprecipitated with CsFT3-like-GFP using GFP-Trap method (Figure 1G). Altogether, it was established that CsFT3-like and CsFD can form a complex to regulate flowering time in saffron, suggesting the CsFT-FD complex module is conserved in saffron.

### 3.2. Transcriptomic Analysis to Identify Genes Regulated by CsFT3-like-FD

**Figure 2.**
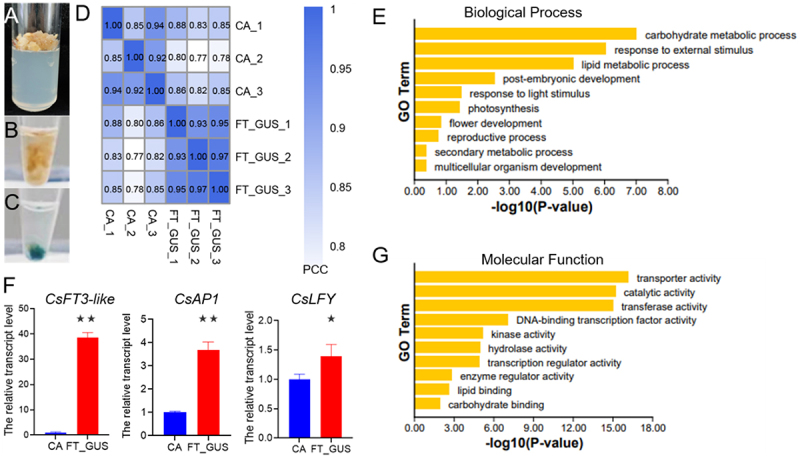
Transcriptome analysis was performed on callus derived from *CsFT3-like*-overexpressing lines. callus derived from the saffron apical buds (A), callus without overexpression of *CsFT3-like* (B) and transgenic lines that successfully expressed *CsFT3-like* (C) were shown. (D) Heatmap correlations of all samples. The degree of similarity between samples increases as the correlation coefficient approaches 1. CA, callus used as the control. FT_GUS, callus overexpressed *CsFT3-like*. PCC, Pearson correlation coefficient. The top 10 significantly enriched gene ontology (GO) terms in biological process (E) and molecular function (F) categories were identified among genes that were upregulated 2-fold in *CsFT3-like*-overexpressing callus. (G) The expression of *CsFT3-like*, *CsAP1* and *CsLFY* was increased in *CsFT3-like*-overexpressing callus. *Tubulin* served as the internal standard. The data are presented as means ± SD, *n* = 3. *p < 0.05 and **p < 0.01.

Following dormancy release, saffron flower buds enter the floral induction phase characterized by vigorous cell division and notable physiological and morphological changes.^29^ Explants obtained from floral buds at this developmental stage were then used for callus induction (Figure 2A, Figure S2), due to their heightened meristematic activity. Since *CsFD* is endogenously expressed in saffron callus (Figure S3), we overexpressed *CsFT3-like* in callus via *Agrobacterium*-mediated transformation to explore the flowering regulatory mechanism mediated by the CsFT3-like-FD complex. Successful transgenic events were validated through GUS histochemical staining (Figure 2B,C). Subsequently, transcriptome sequencing was conducted on three independent biological replicates. Pearson correlation coefficient analysis showed that the samples were well replicated and the data were reliable (Figure 2D).

A total of 13,942 genes differentially expressed (DEGs) were identified, consisting of 7099 down-regulated and 6843 up-regulated genes. Considering the positive regulation of flowering time by the CsFT3-like-FD complex, we focused on up-regulated differentially expressed genes. These genes were significantly enriched in 37 biological processes, with the top 10 processes including carbohydrate metabolism, lipid metabolism, secondary metabolism, response to external and light stimuli, photosynthesis, flower development, post-embryonic development, reproductive processes and multicellular organism development (Figure 2E). Notably, processes of external and light stimulus and flower development were identified as key regulators impacting the initiation of floral primordia and the development of floral organs. These findings highlight the utility of transcriptome analysis in identifying potential candidate genes involved in flowering regulation.

The upregulated differentially expressed genes were enriched in 29 categories of molecular functions, with the top 10 functions primarily including transport activity, catalytic activity, DNA binding transcription factor activity, and transcriptional regulatory activity, etc (Figure 2G). Transcription factors play a crucial role in regulating various plant developmental processes, such as flowering.^38^ To elucidate the mechanisms underlying flowering regulation by the CsFT3-like-FD complex, we focused our analysis on transcription factors. MADS-box and FLO/LFY transcription factors are known to regulate the floral organ specification and floral meristem identity across a range of species. We found that *CsAP1* and *CsLFY* genes were upregulated in callus overexpressing *CsFT3-like*, which was consistent with the verification results of qRT-PCR (Figure 2F). This result indicates that CsFT3-like-FD complex may promote flowering by activating the expression of *CsAP1* and *CsLFY*, which helps to reveal the important role of CsFT-FD complex in the regulation of flowering in saffron.

### 3.3. Genome-Wide Identification of Binding Targets of CsFT3-like-FD

**Figure 3.**
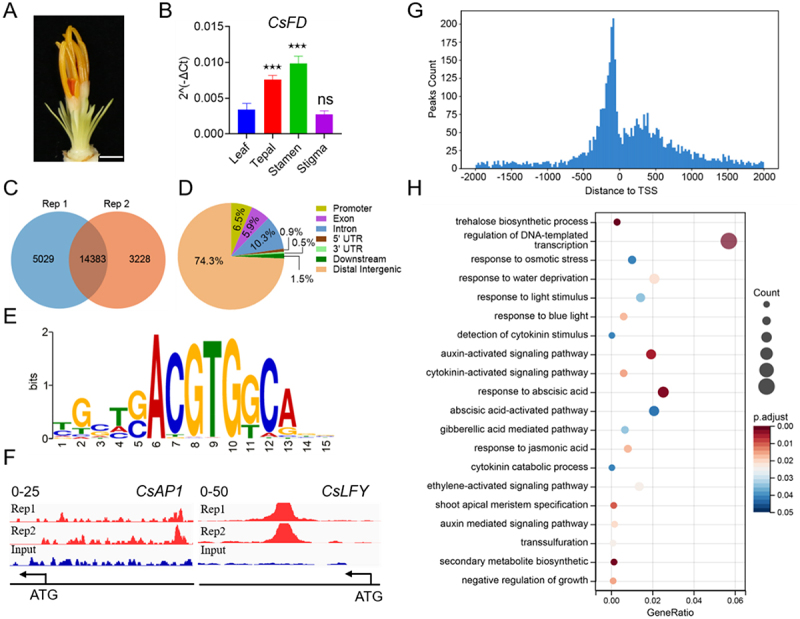
The direct targets across the genome of the CsFT3-like-FD complex were identified using DAP-seq. (A) Saffron floral buds used for DAP-seq were shown. Scale bar represents 0.5 mm. (B) *CsFD* was expressed in leaf, tepal, stamen and stigma. *p < 0.05, **p < 0.01, and ***p < 0.001, ns, no significance. (C) The DAP-seq analysis identified 14,383 high-confidence binding peaks for CsFD. (D) DAP-seq analysis revealed the genome-wide distribution of CsFD-binding peaks. (E) The core DNA sequence of the predominant CsFD-binding motif was shown. (F) Binding profiles for the *CsAP1* and *CsLFY* genes were constructed using data from the input library and the CsFD library. (G) The CsFD binding peaks showed a significant enrichment near the transcription start site (TSS). (H) The top 20 enriched GO biological categories of target genes bound by CsFD are presented. The size of each bubble corresponds to the number of target genes, and the color of the bubble indicates the significance level represented by the *p*-value.

To identify the target genes of CsFT3-like-FD binding, we created gDNA libraries from saffron floral buds (Figure 3A) and conducted DAP-seq to reinforce the transcriptional changes observed in the RNA-Seq dataset. Upon merging the two replicates, a total of 22,640 peaks were detected with a significant enrichment compared to the input libraries (Figure 3C). The peaks were spread across all eight chromosomes of saffron (Figure S4). Upon analyzing their genomic distribution, it was found that the majority of these peaks (74.3%) were situated in distal intergenic regions, with 6.5% in promoter regions, 5.9% in exons, 10.3% in introns, 0.5% in 3’ untranslated regions (UTRs), 0.9% in 5’ UTRs, and 1.5% in downstream regions (Figure 3D). Significantly, a higher number of peaks were identified in the region of −2000 to 2000 bp relative to the transcription start site (TSS) (Figure 3G). We used the MEME-CHIP tool to examine CsFD protein binding motifs, revealing a notable abundance of G-boxes (CACGTG), commonly associated with bZIP proteins (Figure 3E). The GO enrichment analysis of shared targets highlighted their roles in light stimulus responses and various plant hormone signaling pathways, such as cytokinin, auxin, abscisic acid, ethylene, and gibberellic acid-mediated signaling pathways (Figure 3H). Surprisingly, shoot apical meristem specification was also significantly enriched, particularly involving key floral regulators such as *CsAP1* and *CsLFY* (Figure 3F). KEGG pathway analysis further demonstrated notable enrichment in starch and sucrose metabolism (Figure S5). This finding aligns with previous studies that have emphasized the critical role of starch and sucrose metabolism in saffron flowering regulation.^29,39,40^ In a comparison of up-regulated DEGs in *CsFT3-like*-overexpressing saffron callus with candidate target genes from DAP-seq, a total of 3476 genes, including *CsAP1* and *CsLFY*, were identified (Figure S6A). Gene ontology analysis of target genes identified in DAP-seq and RNA-seq showed enrichment in processes related to response to external and endogenous stimuli, flower development, and circadian rhythm (Figure S6B). Taken together, these results indicate that DAP-seq has successfully identified target genes involved in saffron flowering induction processes, such as *CsAP1* and *CsLFY* genes.

### 3.4. CsFT3-like-FD Binds Directly to the CsAp1 and CsLFY Promoters

**Figure 4.**
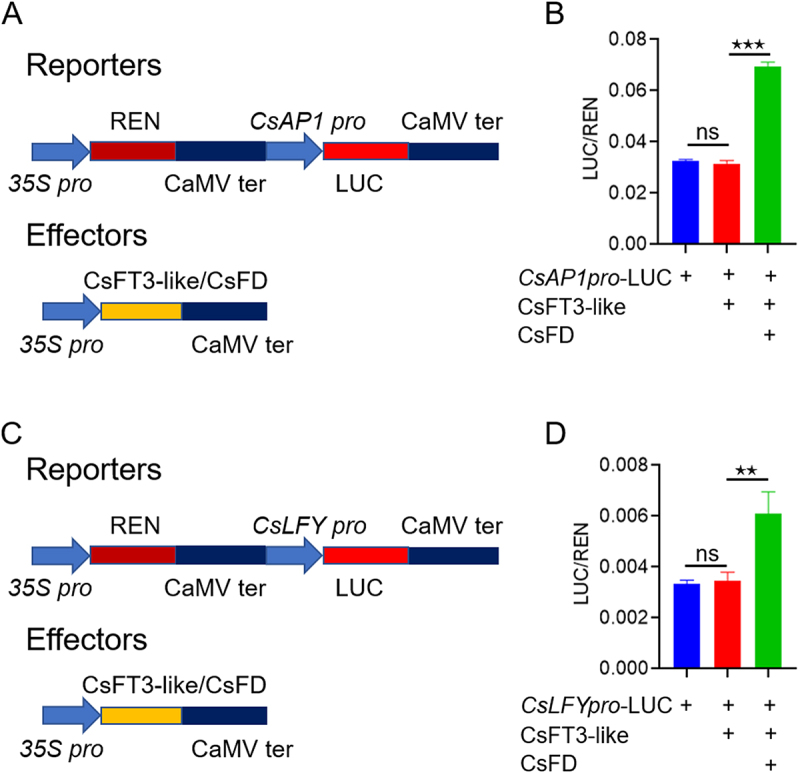
The CsFT3-like-FD complex directly regulates *CsAP1* and *CsLFY*. (A, C) schematic diagrams of the reporter and effector constructs used in dual-luciferase assays. (B, D) the CsFT3-like-FD complex positively regulates the transcriptional activity of the *CsAP1* and *CsLFY*. Luciferase (LUC) activities, normalized to renilla (REN) activities, are shown as mean ± SD of three biological replicates. *p < 0.05, **p < 0.01, and ***p < 0.001, ns, no significance.

*CsLFY* and *CsAP1* were identified as potential targets of CsFT3-like-FD complex through RNA-seq and DAP-seq analysis. Although the DAP-seq data indicate that the CsFT3-like-FD complex binds to the second intron of the *CsLFY* gene (Figure 3F), Dual-LUC assays also show that this complex can bind to the promoter of *CsLFY*. The promoter regions of *CsLFY* and *CsAP1* were cloned into the reporter vector *pGreenII 0800-LUC*, while the coding sequences of *CsFT3-like* and *CsFD* were inserted into the effector vector *pFGC5941* (Figure 4A,C). The reporter and effector vectors were co-expressed in tobacco leaf epidermal cells. The results indicated that the promoters of *CsLFY* and *CsAP1* showed no obvious activation in response to CsFT3-like alone. However, a significant increase in the relative LUC/REN activity was observed upon the co-expression of CsFT3-like and CsFD (Figure 4B,D). This finding suggests that the CsFT3-like-FD complex directly triggers the transcription of *CsAP1* and *CsLFY* genes.

#### Conserved Motifs and Phylogenetic Analysis of CsAP1 and CsLFY Homologs

*AP1* and *LFY* have been reported to determine floral meristem identity and regulate floral organ development in several species.^15,20^ To explore the evolutionary relationships and determine the conservation of function and structure between the saffron CsAP1/CsLFY and their homologs in other angiosperms, we conducted comparative sequence alignment and phylogenetic reconstruction across various species. Since *Crocus sativus* L. is triploid, with three allelic copies of *CsAP1* on chromosome 2 and *CsLFY* on chromosome 8, one allele from each was chosen for further analysis (Figure S7).

**Figure 5.**
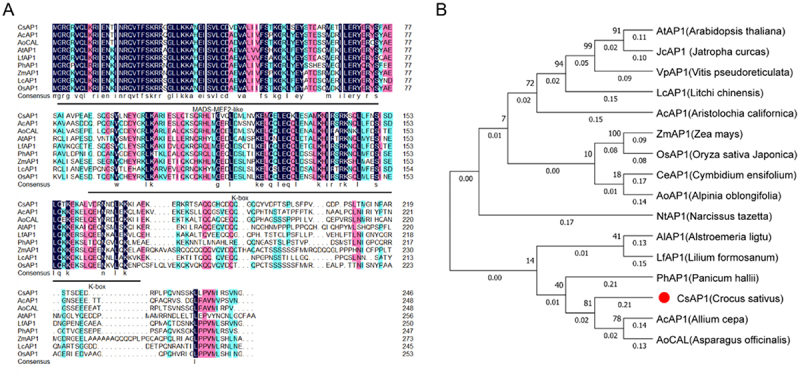
Sequence alignments and phylogenetic tree analysis of CsAP1 and other homologous proteins. (A) Multiple sequence alignments of CsAP1 and eight homologous proteins from other species. The conserved domains are marked by horizontal lines. (B) Phylogenetic analysis of AP1 proteins from different species. These species include *Arabidopsis thaliana*, *jatropha curcas*, *vitis pseudoreticulata*, *litchi chinensis*, *Aristolochia califomica*, *Zea mays*, *oryza sativa Japonica*, *cymbidium ensifolium*, *alpinia oblongifolia*, *narcissus tazetta*, *Alstroemeria ligtu*, *lilium formosanum*, *panicum halli*, *Crocus sativus* L., *alium cepa* and *asparagus officinalis*. The bootstrap value was set to 1000 replicates. The CsAP1 protein is marked with red dot.

The coding sequence of *CsAP1*, which is identical to *CsAP1c*,^41^ spans 741 bp and encodes a protein corresponding to 247 amino acids. Comparative analysis of the amino acid sequences between CsAP1 and AP1 homologs from other species revealed conserved domains, including a canonical MEF2-like MADS domain and a K-box domain (Figure 5A). The phylogenetic tree demonstrates that CsAP1 clusters with monocot AP1 homologs, such as those from *Allium cepa* L. and *Asparagus officinalis* L., while showing relatively distant phylogenetic relationships with dicot AP1 homologs (Figure 5B).

**Figure 6.**
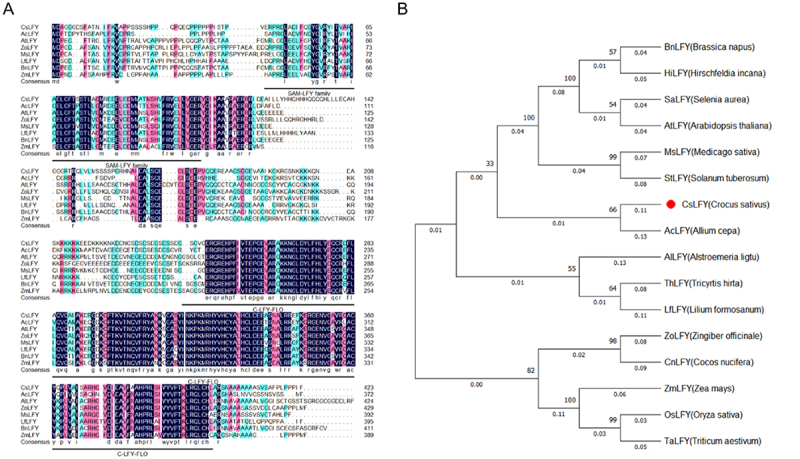
Sequence alignments and phylogenetic tree analysis of CsLFY and other homologous proteins. (A) Multiple sequence alignments of CsLFY and seven homologous proteins from other species. The conserved domains are marked by horizontal lines. (B) Phylogenetic analysis of LFY proteins from other species. These species include *Brassica napus*, *hirschfeldia incana*, *Selenia aurea*, *Arabidopsis thaliana*, *medicago sativa*, *Solanum tuberosum*, *Crocus sativus* L., *Allium cepa*, *Alstroemeria ligtu*, *tricyrtis hirta*, *lilium formosanum*, *zingiber officinale*, *cocos nucifera*, *Zea mays*, *oryza sativa* and *triticum aestivum*. The bootstrap value was set to 1000 replicates. The CsLFY protein is marked with red dot.

The *CsLFY* coding sequence is 1,272 bp in length, encoding a 424 amino acid protein. Comparative analysis of amino acid sequences between CsLFY and LFY homologs from other species revealed highly conserved domains: an N-terminal SAM-LFY superfamily domain and a C-terminal FLO/LFY domain (C-LFY-FLO superfamily) (Figure 6A). The phylogenetic tree showed that CsLFY clusters closely with AcLFY from *Allium cepa* L. (Figure 6B). Collectively, the proteins CsAP1 and CsLFY exhibit similar domains and closer evolutionary relationships to homologs in other species, indicating their conserved roles in plant development, specifically in regulating the floral organ morphogenesis.

### 3.5. Expression Patterns and Subcellular Localization Analysis of CsAP1 and CsLFY

**Figure 7.**
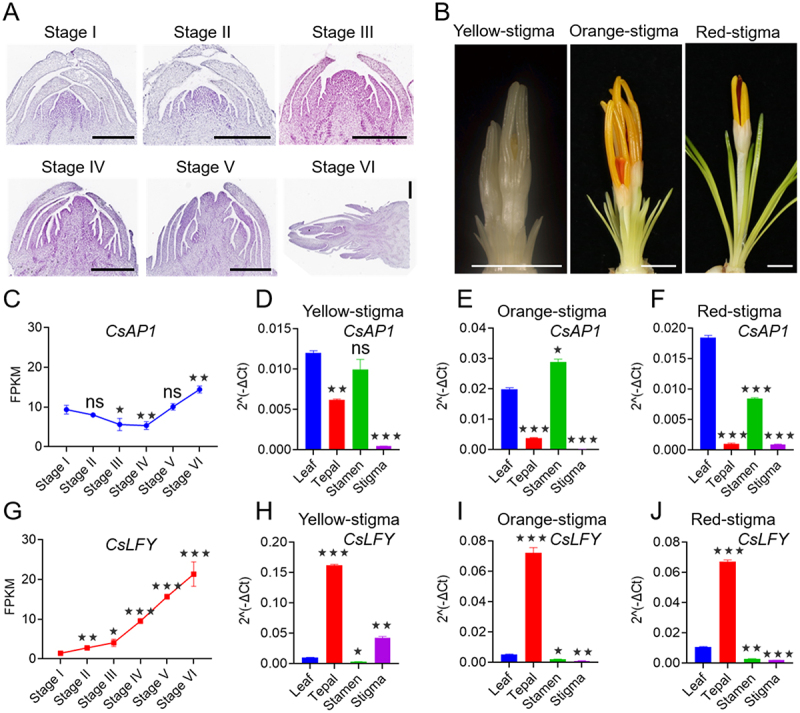
Expression patterns of *CsAP1* and *CsLFY* across different developmental stages of saffron flower buds. (A) Histological paraffin sections of saffron floral buds spanning defined developmental stages, ranging from dormancy to the completion of floral organ differentiation. The lengths of floral buds at different developmental stages are described in the published article by Xi et al. (2024).^29^ Scale bars represent 0.5 mm. (B) Yellow-, orange- and red-stigma stages of floral buds. Scale bars represent 0.5 cm. (C, G) The expression of *CsAP1* and *CsLFY* in floral buds at stages I, ii, iii, IV, V, and VI was presented. (D-F, H-J) The expression levels of *CsAP1* and *CsLFY* in different organs of floral buds at the yellow-, orange-, and red-stigma stages were analyzed by qRT-PCR. The data is represented as the average values from three biological replicates, with error bars denoting the standard deviations. *p < 0.05, **p < 0.01, and ***p < 0.001, ns, no significance.

The flowering process in saffron is protracted, mainly including dormancy, flower induction, flower organ differentiation and blooming.^42,43^ To study the tissue-specific expression patterns of *CsAP1* and *CsLFY*, we analyzed the transcriptome data (https://www.ncbi.nlm.nih.gov/, PRJNA1206159) on flower bud development in saffron. Our findings revealed that the expression level of *CsAP1* remained relatively stable from the dormancy stage to the end of flower primordium formation (Stage I to Stage IV)^29^ (Figure 7A), with a gradual increase observed as the flower bud transitioned into the flower organ differentiation stage (Stage IV to Stage V) (Figure 7C). During the growth of floral organs in saffron, this process is primarily categorized into three stages: yellow-, orange-, and red-stigma stages (Figure 7B). *CsAP1* exhibits predominant expression in the leaves, tepals, and stamens throughout these stages, with some minor expression also observed in the stigmas (Figure 7D–F). After dormancy is lifted, saffron floral buds go through the floral induction stage (Stage II-IV). During this stage, *CsLFY* begins to rise gradually and continues until floral organ differentiation is completed (Stage IV-VI) (Figure 7G). The expression pattern of *CsLFY* during saffron floral development revealed predominant transcript accumulation in leaves and tepals throughout the yellow-, orange-, and red-stigma stages (Figure 7H–J). In contrast, significantly lower expression was detected in stamens and stigmas.

**Figure 8.**
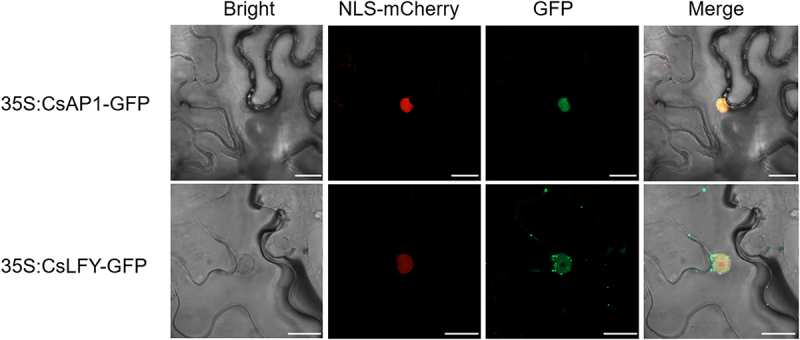
The subcellular localization of CsAP1 and CsLFY proteins. The constructs of *35S:CsAP1-GFP* and 3*5S:CsLFY-GFP* were transiently co-expressed with the nuclear marker *35S:SV40NLS-mCherry* in the epidermal cells of *Nicotiana benthamiana* leaves via *Agrobacterium*-mediated transformation. Scale bars represent 20 µm.

To investigate the subcellular localization of CsAP1 and CsLFY proteins, the fusion constructs *pro35S:CsAP1-GFP* and *pro35S:CsLFY-GFP* were co-expressed in tobacco leaf epidermal cells with a nuclear marker *pro35S:NLS-mCherry*. As shown in Figure 8, the green fluorescence signals of CsAP1-GFP and CsLFY-GFP completely coincided with the red fluorescence signals of the nuclear marker (NLS-mCherry), demonstrating that CsAP1 and CsLFY are both nuclear-localized proteins.

### 3.6. *CsAP1* and *CsLFY* Promote Flowering, with *CsAP1* Affecting Floral Organ

**Figure 9.**
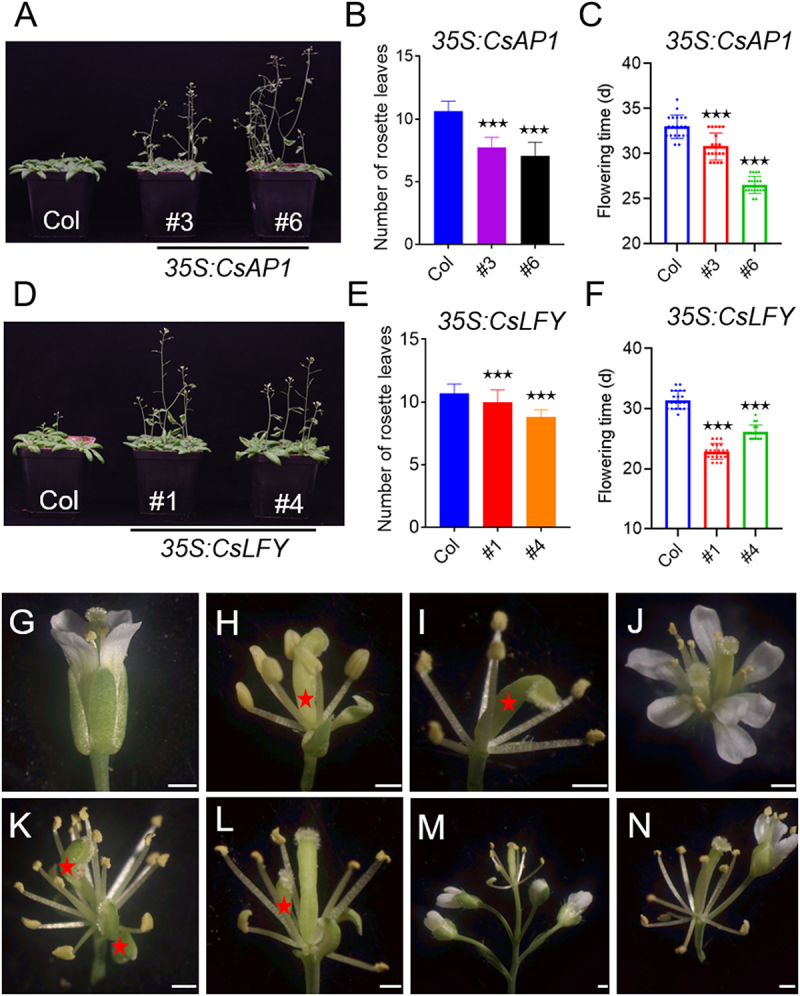
The phenotypes of ectopic expression of *CsAP1* and *CsLFY* in *Arabidopsis thaliana* were shown. ectopic expression of *CsAP1* (A) and *CsLFY* (D) in *Arabidopsis* induces an early flowering phenotype under long day conditions. Comparison of the counts of rosette leaves in wt, *CsAP1* overexpressing plants (B) and *CsLFY* overexpressing plants (E) at flowering. Comparison of flowering time in WT, *CsAP1* overexpressing plants (C) and *CsLFY* overexpressing plants (F). The data are presented as means ± SD. *p < 0.05, **p < 0.01, ***p < 0.001. (G) The flower of WT plants. (H-N) Overexpressing *CsAP1* causes homeotic transformations in floral organs. (H) Flowers without tepals, with only two sepals and curved pistils. (I) Flowers lacking both tepals and sepals, and featuring curved pistils. (J) Flowers with increased tepals, stamens and pistils. (K) Flowers with more stamens and pistils but no tepals or sepals. (L) Flowers with increased numbers of stamens and pistils, no tepals, and two sepals. (M, N) Flowers with an increased number of stamens, no tepals or sepals, but with normally developed pistils. The red arrows represent the pistils. Scale bars represent 0.5 mm.

Based on multiple sequence alignments and phylogenetic tree analysis, CsAP1 and CsLFY likely perform similar functions to AP1 and LFY proteins from other species. To investigate the potential function of *CsAP1* and *CsLFY* in floral meristem transition and organ identity specification, transgenic *Arabidopsis* lines overexpressing *pro35S:CsAP1* and *pro35S:CsLFY* constructs were generated (Figure S8A). We conducted an analysis of flowering time and floral organ phenotypes of homozygous transgenic lines under long-day conditions. Ectopic expression of *CsAP1* and *CsLFY* in *Arabidopsis* (Col-0) significantly accelerated flowering (Figure 9A–F).

To further investigate whether *CsAP1* and *CsLFY* overexpression alters the expression of flowering-related genes in *Arabidopsis*, qPCR assays were conducted to assess the endogenous gene *AtFT*, *AtMFT*, *AtTSF*, *AtAP1*, *AtLFY* and *AtSOC1*. The results demonstrated that the expression of *AtFT*, *AtAP1* and *AtSOC1* was markedly upregulated in *pro35S:CsAP1* plants that only exhibited the early-flowering phenotype (Figure S8B). In contrast, *pro35S:CsLFY* plants showed upregulation in the expression of *AtMFT*, *AtAP1*, and *AtLFY* (Figure S8C). In *Arabidopsis*, *AtLFY* and *AtAP1* act antagonistically to regulate several key flowering-related genes, such as *AtTFL1*, *AtFD*, *AtTEM1* and *AtAP2*.^27^ However, in our *CsAP1*- and *CsLFY*-overexpressing lines, most of these downstream genes did not show opposing expression trends (Figure S9). Interestingly, *AtFT* expression exhibited an inverse pattern between the two overexpression lines (Figure S8B, C). These observations suggest that the functions of homologous genes may vary across different species.

The overexpression of *CsAP1* in *Arabidopsis thaliana* resulted in conspicuous flower organ defects in 60% of transgenic plants. Strikingly, 30% of these plants displayed tepals were absent, only stamens and pistils remained, and the number of sepals was reduced to 0–2 (Figure 9H,I). 62% of the plants showed an increase in tepals, stamens and pistils (Figure 9J–L). 67% of the plants exhibited a unique pattern with more stamens but a complete absence of tepals and sepals (Figure 9M,N). Intriguingly, there was a notable increase in the percentage of pistil weight to the total flower weight (Figure S10), making it a promising target for breeding elite *C. sativus* varieties with enhanced stigma yield. Taken together, these results demonstrate that both *CsAP1* and *CsLFY* serve as crucial regulators in the genetic network governing flowering time and flower morphology.

## 4. Discussion

The precise regulation of plant flowering time arises from the synergistic integration of environmental stimuli and endogenous factors.^44,45^ The intricate regulatory network has been extensively characterized in model plant species, such as *Arabidopsis thaliana* and *Oryza sativa*. Saffron, a triploid asexual species, possesses a complex genome and a limited genetic transformation system, making the exploration of its flowering regulation mechanism quite challenging. Current research on saffron floral induction has primarily focused on environmental factors such as light, temperature, and phytohormones,^43,46–48^ while molecular-level studies have largely been limited to omics analyses.^39,40,42^ Recently, Kalia et al., 2025^5^ reported that CsatFT3 and CsatTFL1-3 compete for binding to CsatFD2, with their balance regulating floral induction, a finding that has significantly advanced research in the field of saffron. In this study, we demonstrate that CsFT3-like interacts with a novel CsFD protein to form a complex that directly regulates expression of the floral meristem identity genes *CsAP1* and *CsLFY*. These findings offer an alternative perspective on the molecular mechanisms governing floral induction in saffron.

### 4.1. CsFT3-like and CsFD Interact with Each Other to Form a Complex in Saffron

The mechanism by which FT interacts with FD to form complexes that activate flowering-related genes and promote flowering is conserved in plants, as demonstrated in *Arabidopsis*,^49^ rice,^50^ and maize.^51^ Recent work has extended this framework to saffron. Kalia et al. (2025)^5^ showed that CsatFT3 binds CsatFD2 and competes with CsatTFL1–3/CEN1 to regulate floral induction. We further identified a novel CsFD protein that interacts with CsFT3-like. However, the functional mechanisms of the CsFT3-like–CsFD complex remain to be elucidated.

Overexpressing *CsFT*3*-like* and *CsFD* in wild-type *Arabidopsis* resulted in early flowering phenotypes, indicating their conserved roles in floral transition. Yeast two-hybrid and co-immunoprecipitation assays confirmed that CsFT3-like and CsFD interact with each other. Furthermore, both genes were expressed in saffron flower buds during the floral induction stage, with subcellular localization assays confirming their nuclear enrichment. These findings provide the possibility that CsFT3-like and CsFD form a complex to regulate flowering-related genes and promote floral induction.

### 4.2. The CsFT3-like-FD Complex Promotes Flowering by Directly Regulating CsAP1 and CsLFY

**Figure 10.**
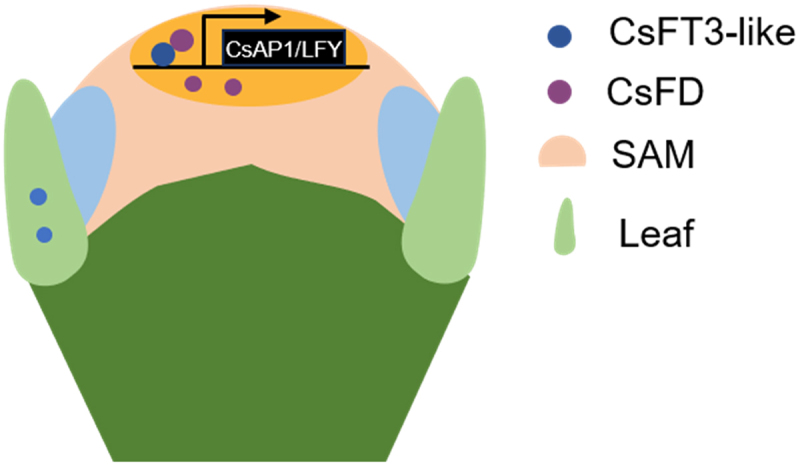
The model of the CsFT3-like-FD complex regulating the floral transition in saffron. at the shoot apical meristem (SAM) of saffron flower buds, the CsFT3-like protein interacts with CsFD to form a functional complex. This complex then directly activates the transcription of the floral meristem identity genes *CsAP1* and *CsLFY*, triggering the transition to flowering.

We initially analyzed RNA-seq data from *CsFT3-like*-overexpressing saffron callus to identify differentially expressed genes across multiple functional categories. Considering the pivotal role of transcription factors in integrating plant organ morphogenesis, cell fate determination, and environmental signal perception,^52,53^ we focused on CsAP1 and CsLFY as key candidate regulators. Subsequent DAP-seq profiling of the CsFD protein revealed genome-wide binding sites, with the identification of a conserved G-box motif (5‘-CACGTG-3‘), a cis-element previously characterized in *Arabidopsis* FD protein.^54^ Notably, the G-box sequences were detected in *CsAP1* and *CsLFY* genes, implying their direct regulation by the CsFT3-like-FD complex. Dual-LUC assays confirmed that the CsFT3-like-FD complex directly activates the transcription of *CsAP1* and *CsLFY*. Further investigations showed that *CsAP1* and *CsLFY* are also expressed in flower buds during the floral induction stage and exhibit nuclear localization patterns consistent with those of CsFT3-like and CsFD. These results support the model that CsFT3-like and CsFD form a complex in saffron floral buds to activate *CsAP1* and *CsLFY*, thereby promoting flowering (Figure 10).

### 4.3. The CsFT3-like-FD-AP1/LFY Module Regulates Flowering Time and Floral Organ Development

Notably, the three *CsatAP1/FUL* genes in *Crocus* were first isolated and classified as E-class genes within the ABCDE model.^41,55^ RT-PCR analysis revealed a broad accumulation of steady-state mRNA for all three homologs not only across all mature floral organs but also in leaves. This expression pattern differs from that of *Arabidopsis AP1*, which is specifically expressed in tissues determining floral fate and is restricted to sepals and petals within the flower.^41^ Furthermore, while *AtAP1* and many of its dicot orthologs contain a C-terminal CFAA motif, a canonical CaaX box recognized by farnesyltransferase (FTase) for regulating *AP1* function and potentially its specificity, the three *CsAP1* genes in *Crocus* lack this conserved motif.^56^ Taken together, their distinct expression profile and the absence of the conserved CaaX box suggest that the *CsAP1* genes may have functionally diverged from the typical *Arabidopsis AP1* model.

To explore the biological functions of *CsAP1* and *CsLFY*, we generated *Arabidopsis* transgenic lines with constitutively overexpressing these genes. Phenotypic analysis revealed that both transgenic lines exhibited significantly earlier flowering compared to wild-type plants, confirming their evolutionarily conserved roles in floral promotion. Particularly, *CsAP1*-overexpressing lines displayed characteristic homeotic transformations of floral organs, primarily manifested as altered numbers of tepals, sepals, stamens, and pistils. This observation aligns with previous findings that overexpression of *Osmanthus fragrans OfAP1* in *Arabidopsis* similarly caused abnormal floral organ development.^19^ In *Arabidopsis*, overexpression of *AtAP1* accelerates flowering and converts inflorescence meristems into floral meristems, sometimes even producing solitary flowers. Conversely, disruption of *AP1* function leads to abnormal floral organ development.^57,58^ These findings collectively demonstrate that the functions of *AP1* orthologs are partially conserved across different species but also show evidence of functional divergence.

Extending the flowering period of saffron holds considerable theoretical promise for enhancing crop yield. The economic value of saffron is largely derived from the valuable stigmas of its flowers. However, under natural conditions, each individual flower has a very short bloom duration, and the overall flowering window is highly narrow. A prolonged flowering period could mitigate harvesting constraints, reduce vulnerability to adverse environmental conditions, and potentially increase the total yield of harvestable stigmas by enabling multiple flowering cycles per plant within a single growing season.^59^ Our preliminary investigations indicate that the *CsAP1* and *CsLFY* genes can substantially modulate flowering time in model plants, offering promising candidate genetic resources for improving saffron yield through targeted regulation of its flowering phenology. Stigma weight is a crucial factor in determining saffron yield, as the commercial value of this medicinal crop derives predominantly from its stigmas. Unlike the overexpression of *CsLFY*, which showed no significant effect on the ratio of pistil weight to total flower weight compared to the wild type, the *CsAP1* overexpression transgenic lines exhibited a marked increase in this proportion (Figure S10). Thus, these findings collectively indicate that *CsAP1* serves not only as a regulator of flowering time but may also reprogram assimilate partitioning toward the economically essential stigma, thereby providing a dual-target genetic strategy to improve the yield in saffron.

## 5. Conclusions

In summary, this study highlights the pivotal role of the CsFT3-like-FD-AP1/LFY regulatory module in saffron flowering. *CsLFY* and *CsAP1* were identified as potential targets of CsFT3-like-FD complex through RNA-seq and DAP-seq analysis. Direct transcriptional activation of *CsAP1* and *CsLFY* by this complex was further confirmed through Dual–LUC assays. Spatial expression analysis revealed that *CsAP1* is predominantly expressed in the leaves, tepals, and stamens of saffron, whereas *CsLFY* is mainly expressed in the leaves and tepals. Both CsAP1 and CsLFY are localized in the nucleus. Ectopic expression of *CsAP1* and *CsLFY* in *Arabidopsis* significantly promotes flowering, with *CsAP1* overexpression additionally causing floral organ developmental abnormalities.

## Supplementary Material

Supplemental Material

Flowering Time Data.xlsx

## Data Availability

The datasets supporting the conclusions of this article are included within the article.

## References

[1] Cardone L, Castronuovo D, Perniola M, Cicco N, Candido V. Saffron (Crocus sativus L.), the king of spices: an overview. Sci Hortic. 2020;272:109560. doi: 10.1016/j.scienta.2020.109560.

[2] Abu-Izneid T, Rauf A, Khalil AA, Olatunde A, Khalid A, Alhumaydhi FA, Aljohani ASM, Sahab Uddin M, Heydari M, Khayrullin M, et al. Nutritional and health beneficial properties of saffron (Crocus sativus L): a comprehensive review. Crit Rev Food Sci Nutr. 2022;62(10):2683–18. doi: 10.1080/10408398.2020.1857682. 33327732

[3] Yang W, Qiu X, Wu Q, Chang F, Zhou T, Zhou M, Pei J. Active constituents of saffron (Crocus sativus L.) and their prospects in treating neurodegenerative diseases (review). Exp Ther Med. 2023;25(5):235. doi: 10.3892/etm.2023.11934. 37114174 PMC10127217

[4] Wang F, Li S, Kong F, Lin X, Lu S. Altered regulation of flowering expands growth ranges and maximizes yields in major crops. Front Plant Sci. 2023;14:1094411. doi: 10.3389/fpls.2023.1094411. 36743503 PMC9892950

[5] Kalia D, Jose-Santhi J, Sheikh FR, Singh RK. Florigen activation complex dynamics and SVP-mediated repression orchestrate temperature-regulated flowering in saffron. Plant Biotechnol J. 2025; 1–19. doi: 10.1111/pbi.70361. PMC12946510 41078107

[6] Zhou T, Qiu X, Zhao L, Yang W, Wen F, Wu Q, Pei J, Xu B, Chen J, Ma Y, et al. Optimal light intensity and quality increased the saffron daughter corm yield by inhibiting the degradation of reserves in mother corms during the reproductive stage. Ind Crops Prod. 2022;176:114396. doi: 10.1016/j.indcrop.2021.114396.

[7] Gao H, Ding N, Wu Y, Yu D, Zhou SZ, Stolze SC, Vincent C, Rodríguez Maroto G, de Los Reyes P, Harzen A, et al. Florigen activation complex forms via multifaceted assembly in Arabidopsis. Nature. 2025;648(8094):686–95. doi: 10.1038/s41586-025-09704-6. 41225013 PMC12711580

[8] Jiang X, Lubini G, Hernandes-Lopes J, Rijnsburger K, Veltkamp V, de Maagd RA, Angenent GC, Bemer M. Fruitfull-like genes regulate flowering time and inflorescence architecture in tomato. Plant Cell. 2022;34(3):1002–19. doi: 10.1093/plcell/koab298. 34893888 PMC8894982

[9] Jung JH, Lee HJ, Ryu JY, Park CM. Spl3/4/5 integrate developmental aging and photoperiodic signals into the FT-FD module in Arabidopsis flowering. Mol Plant. 2016;9(12):1647–59. doi: 10.1016/j.molp.2016.10.014. 27815142

[10] Park KH, Kim SB, Jung JH. Analysis of temperature effects on the protein accumulation of the FT-FD module using newly generated Arabidopsis transgenic plants. Plant Direct. 2023;7(12):e552. doi: 10.1002/pld3.552. 38116182 PMC10727963

[11] Tylewicz S, Tsuji H, Miskolczi P, Petterle A, Azeez A, Jonsson K, Shimamoto K, Bhalerao RP. Dual role of tree florigen activation complex component FD in photoperiodic growth control and adaptive response pathways. Proc Natl Acad Sci USA. 2015;112(10):3140–45. doi: 10.1073/pnas.1423440112. 25713384 PMC4364234

[12] Zhang P, Liu H, Mysore KS, Wen J, Meng Y, Lin H, Niu L. Mtfda is essential for flowering control and inflorescence development in Medicago truncatula. J Plant Physiol. 2021;260:153412. doi: 10.1016/j.jplph.2021.153412. 33845341

[13] Kaufmann K, Melzer R, Theissen G. Mikc-type mads-domain proteins: structural modularity, protein interactions and network evolution in land plants. Gene. 2005;347(2):183–98. doi: 10.1016/j.gene.2004.12.014. 15777618

[14] Murai K. Homeotic genes and the ABCDE model for floral organ formation in wheat. Plants. 2013;2(3):379–95. doi: 10.3390/plants2030379. 27137382 PMC4844379

[15] Kaufmann K, Wellmer F, Muiño JM, Ferrier T, Wuest SE, Kumar V, Serrano-Mislata A, Madueño F, Krajewski P, Meyerowitz EM, et al. Orchestration of floral initiation by APETALA1. Science. 2010;328(5974):85–89. doi: 10.1126/science.1185244. 20360106

[16] Bowman JL, Alvarez J, Weigel D, Meyerowitz EM, Smyth DR. Control of flower development in Arabidopsis thaliana by APETALA1 and interacting genes. Development. 1993;119(3):721–43. doi: 10.1242/dev.119.3.721.

[17] Chen L, Nan H, Kong L, Yue L, Yang H, Zhao Q, Fang C, Li H, Cheng Q, Lu S, et al. Soybean ap1 homologs control flowering time and plant height. J Integr Plant Biol. 2020;62(12):1868–79. doi: 10.1111/jipb.12988. 32619080

[18] Chi Y, Huang F, Liu H, Yang S, Yu D. An APETALA1-like gene of soybean regulates flowering time and specifies floral organs. J Plant Physiol. 2011;168(18):2251–59. doi: 10.1016/j.jplph.2011.08.007. 21963279

[19] Liu X, Wang Q, Jiang G, Wan Q, Dong B, Lu M, Deng J, Zhong S, Wang Y, Khan IA, et al. Temperature-responsive module of OfAP1 and OfLFY regulates floral transition and floral organ identity in Osmanthus fragrans. Plant Physiol Biochem. 2023;203:108076. doi: 10.1016/j.plaphy.2023.108076. 37832366

[20] Zhao W, Chen Z, Liu X, Che G, Gu R, Zhao J, Wang Z, Hou Y, Zhang X. CsLFY is required for shoot meristem maintenance via interaction with WUSCHEL in cucumber (Cucumis sativus). New Phytol. 2018;218(1):344–56. doi: 10.1111/nph.14954. 29274285

[21] Hu J, Jin Q, Ma Y. AfLFY, a leafy homolog in Argyranthemum frutescens, controls flowering time and leaf development. Sci Rep. 2020;10(1):1616. doi: 10.1038/s41598-020-58570-x. 32005948 PMC6994665

[22] Weigel D, Alvarez J, Smyth DR, Yanofsky MF, Meyerowitz EM. Leafy controls floral meristem identity in Arabidopsis. Cell. 1992;69(5):843–59. doi: 10.1016/0092-8674(92)90295-n. 1350515

[23] Blázquez MA, Soowal LN, Lee I, Weigel D. Leafy expression and flower initiation in Arabidopsis. Development. 1997;124(19):3835–44. doi: 10.1242/dev.124.19.3835. 9367439

[24] Jue D, Li Z, Zhang W, Tang J, Xie T, Sang X, Guo Q. Identification and functional analysis of the LEAFY gene in longan flower induction. BMC Genomics. 2024;25(1):308. doi: 10.1186/s12864-024-10229-x. 38528464 PMC10962150

[25] Liu Y, Zhao Q, Meng N, Song H, Li C, Hu G, Zhang Z, Lin S, Zhang Z. Over-expression of EjLFY-1 leads to an early flowering habit in strawberry (Fragaria×ananassa) and its asexual progeny. Front Plant Sci. 2017;8:496. doi: 10.3389/fpls.2017.00496. 28443106 PMC5385365

[26] Rao NN, Prasad K, Kumar PR, Vijayraghavan U. Distinct regulatory role for RFL, the rice LFY homolog, in determining flowering time and plant architecture. Proc Natl Acad Sci USA. 2008;105(9):3646–51. doi: 10.1073/pnas.0709059105. 18305171 PMC2265177

[27] Goslin K, Zheng B, Serrano-Mislata A, Rae L, Ryan PT, Kwaśniewska K, Thomson B, Ó’Maoiléidigh DS, Madueño F, Wellmer F, et al. Transcription factor interplay between LEAFY and APETALA1/CAULIFLOWER during floral initiation. Plant Physiol. 2017;174(2):1097–109. doi: 10.1104/pp.17.00098. 28385730 PMC5462026

[28] Serrano-Mislata A, Goslin K, Zheng B, Rae L, Wellmer F, Graciet E, Madueño F. Regulatory interplay between leafy, APETALA1/CAULIFLOWER and TERMINAL FLOWER1: new insights into an old relationship. Plant Signal Behav. 2017;12(10):e1370164. doi: 10.1080/15592324.2017.1370164. 28873010 PMC5647955

[29] Xi X, Li J, Song J, Qian X, Xu X, Feng M, Li L. CsERECTA alternative splicing regulates the flowering numbers depending on temperature in Crocus sativus L. Ind Crop Prod. 2024;218:118971. doi: 10.1016/j.indcrop.2024.118971.

[30] Clough SJ, Bent AF. Floral dip: a simplified method for Agrobacterium-mediated transformation of Arabidopsis thaliana. Plant J. 1998;16(6):735–43. doi: 10.1046/j.1365-313x.1998.00343.x. 10069079

[31] Chib S, Thangaraj A, Kaul S, Dhar MK, Kaul T. Development of a system for efficient callus production, somatic embryogenesis and gene editing using CRISPR/Cas9 in saffron (Crocus sativus L.). Plant Methods. 2020;16(1):47. doi: 10.1186/s13007-020-00589-2. 32280363 PMC7137501

[32] Xi X, Hu Z, Nie X, Meng M, Xu H, Li J. Cross inhibition of MPK10 and WRKY10 participating in the growth of endosperm in Arabidopsis thaliana. Front Plant Sci. 2021;12:640346. doi: 10.3389/fpls.2021.640346. 33897728 PMC8062763

[33] Hellens RP, Allan AC, Friel EN, Bolitho K, Grafton K, Templeton MD, Karunairetnam S, Gleave AP, Laing WA. Transient expression vectors for functional genomics, quantification of promoter activity and RNA silencing in plants. Plant Methods. 2005;1(1):13. doi: 10.1186/1746-4811-1-13. 16359558 PMC1334188

[34] Li L, He J, Qian X, Xi X, Li J, Chen J, Yang S, Tao Y, Feng M, Zhang X, et al. A resource for improving the quality and yield of Crocus sativus stigma. Ind Crops Prod. 2025;237:122141. doi: 10.1016/j.indcrop.2025.122141.

[35] Bai D, Zhong Y, Gu S, Qi X, Sun L, Lin M, Wang R, Li Y, Hu C, Fang J. AvERF73 positively regulates waterlogging tolerance in kiwifruit by participating in hypoxia response and mevalonate pathway. Hortic Plant J. 2025;11(1):162–74. doi: 10.1016/j.hpj.2023.05.021.

[36] Zhang W, Tang S, Li X, Chen Y, Li J, Wang Y, Bian R, Jin Y, Zhu X, Zhang K. Arabidopsis wrky1 promotes monocarpic senescence by integrative regulation of flowering, leaf senescence, and nitrogen remobilization. Mol Plant. 2024;17(8):1289–306. doi: 10.1016/j.molp.2024.07.005. 39003499

[37] Tsaftaris A, Pasentsis K, Argiriou A. Cloning and characterization of flowering locus T-like genes from the perennial geophyte saffron crocus (Crocus sativus). Plant Mol Biol Rep. 2013;31(6):1558–68. doi: 10.1007/s11105-013-0608-x.

[38] Zou X, Sun H. Dof transcription factors: specific regulators of plant biological processes. Front Plant Sci. 2023;14:1044918. doi: 10.3389/fpls.2023.1044918. 36743498 PMC9897228

[39] Chen J, Zhou G, Dong Y, Qian X, Li J, Xu X, Huang H, Xu L, Li L. Screening of key proteins affecting floral initiation of saffron under cold stress using iTRAQ-based proteomics. Front Plant Sci. 2021;12:644934. doi: 10.3389/fpls.2021.644934. 34046047 PMC8144468

[40] Jose-Santhi J, Sheikh FR, Kalia D, Singh RK. Sugar metabolism mediates temperature-dependent flowering induction in saffron (Crocus sativus L.). Environ Exp Bot. 2023;206:105150. doi: 10.1016/j.envexpbot.2022.105150.

[41] Tsaftaris A, Pasentsis K, Iliopoulos I, Polidoros A. Isolation of three homologous AP1-like MADS-box genes in crocus (Crocus sativus L.) and characterization of their expression. Plant Sci. 2004;166(5):1235–43. doi: 10.1016/j.plantsci.2003.12.037.

[42] Renau-Morata B, Nebauer SG, García-Carpintero V, Cañizares J, Minguet EG, De Los Mozos M, Molina RV. Flower induction and development in saffron: timing and hormone signalling pathways. Ind Crops Prod. 2021;164:113370. doi: 10.1016/j.indcrop.2021.113370.

[43] Wang Z, Li X, Xu J, Yang Z, Zhang Y. Effects of ambient temperature on flower initiation and flowering in saffron (Crocus sativus). Sci Hortic. 2021;279:109859. doi: 10.1016/j.scienta.2020.109859.

[44] Freytes SN, Canelo M, Cerdán PD. Regulation of flowering time: when and where? Curr Opin Plant Biol. 2021;63:102049. doi: 10.1016/j.pbi.2021.102049. 33975153

[45] Wang J, Wang Q, Gao J, Lei Y, Zhang J, Zou J, Yang W, Li S, Lei N, Dhungana D, et al. Genetic regulatory pathways of plant flowering time affected by abiotic stress. Plant Stress. 2025;100747:100747. doi: 10.1016/j.stress.2025.100747.

[46] Li L, Zhou Y, Wang J, Qi X, Fang H, Bai Y, Chen Z, Yu X, Liu D, Liu Q, et al. Effects of supplementary light treatment on saffron: integrated physiological, metabolomic, and transcriptome analyses. BMC Plant Biol. 2024;24(1):1247. doi: 10.1186/s12870-024-05944-2. 39722040 PMC11670385

[47] Molina RV, Valero M, Navarro Y, Guardiola JL, Garcia-Luis AJSH. Temperature effects on flower formation in saffron (Crocus sativus L.). Sci Hortic. 2005;103(3):361–79. doi: 10.1016/j.scienta.2004.06.005.

[48] Singh D, Sharma S, Jose-Santhi J, Kalia D, Singh RK. Hormones regulate the flowering process in saffron differently depending on the developmental stage. Front Plant Sci. 2023;14:1107172. doi: 10.3389/fpls.2023.1107172. 36968363 PMC10034077

[49] Abe M, Kobayashi Y, Yamamoto S, Daimon Y, Yamaguchi A, Ikeda Y, Ichinoki H, Notaguchi M, Goto K, Araki T. Fd, a bZIP protein mediating signals from the floral pathway integrator FT at the shoot apex. Science. 2005;309(5737):1052–56. doi: 10.1126/science.1115983. 16099979

[50] Taoka K, Ohki I, Tsuji H, Furuita K, Hayashi K, Yanase T, Yamaguchi M, Nakashima C, Purwestri YA, Tamaki S, et al. 14-3-3 proteins act as intracellular receptors for rice Hd3a florigen. Nature. 2011;476(7360):332–35. doi: 10.1038/nature10272. 21804566

[51] Sun H, Wang C, Chen X, Liu H, Huang Y, Li S, Dong Z, Zhao X, Tian F, Jin W. Dlf1 promotes floral transition by directly activating ZmMADS4 and ZmMADS67 in the maize shoot apex. New Phytol. 2020;228(4):1386–400. doi: 10.1111/nph.16772. 32579713

[52] Koyama T. Regulatory mechanisms of transcription factors in plant morphology and function. Int J Mol Sci. 2023;24(8):7039. doi: 10.3390/ijms24087039. 37108201 PMC10138701

[53] Strader L, Weijers D, Wagner D. Plant transcription factors - being in the right place with the right company. Curr Opin Plant Biol. 2022;65:102136. doi: 10.1016/j.pbi.2021.102136. 34856504 PMC8844091

[54] Izawa T, Foster R, Chua NH. Plant bZIP protein DNA binding specificity. J Mol Biol. 1993;230(4):1131–44. doi: 10.1006/jmbi.1993.1230. 8487298

[55] Tsaftaris SA, Kalivas A, Pasentsis K, Argiriou A. Expression analysis of flower MADS-box genes in saffron Crocus (Crocus sativus L.) supports a modified ABCDE model. Saffron. Global Sci Books. 2010;UK/Japan:38–44.

[56] Tsaftaris A, Polidoros A, Pasentsis K, Kalivas A. Cloning, structural characterization, and phylogenetic analysis of flower MADS-box genes from crocus (Crocus sativus L.). Sci World J. 2007;7:1047–62. doi: 10.1100/tsw.2007.175. PMC5900851 17619787

[57] Irish VF, Sussex IM. Function of the apetala-1 gene during Arabidopsis floral development. Plant Cell. 1990;2(8):741–53. doi: 10.1105/tpc.2.8.741. 1983792 PMC159927

[58] Mandel MA, Yanofsky MF. A gene triggering flower formation in Arabidopsis. Nature. 1995;377(6549):522–24. doi: 10.1038/377522a0. 7566148

[59] Molina RV, García-Luis A, Valero M, Navarro Y, Guardiola JL. Extending the harvest period of saffron. Acta Hortic. 2004;650(650):219–25. doi: 10.17660/ActaHortic.2004.650.25.

