# Supplementary material for: The CsFT-FD complex controls flowering by regulating CsAP1 and CsLFY in saffron
Source: GM Crops Food. 2026 Apr 1;17(1):2651568. doi: 10.1080/21645698.2026.2651568 (PMC13048584; doi:10.1080/21645698.2026.2651568)
Supplement: Supplemental Material [file KGMC_A_2651568_SM9589.docx]

**SUPPLEMENTARY MATERIAL**


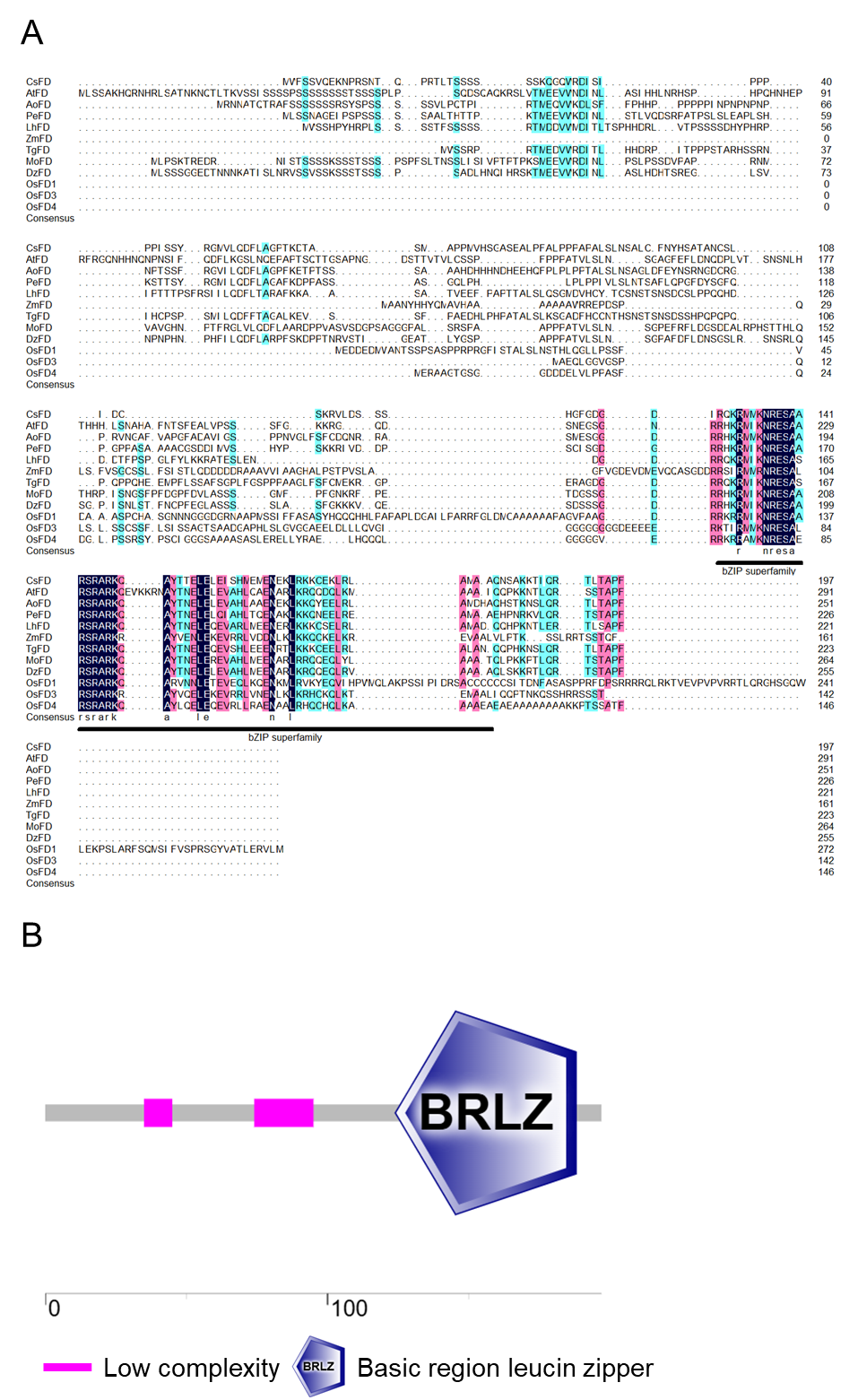


**Fig.S1 Multiple sequence alignments (A) and protein domain prediction (B) of CsFD.**


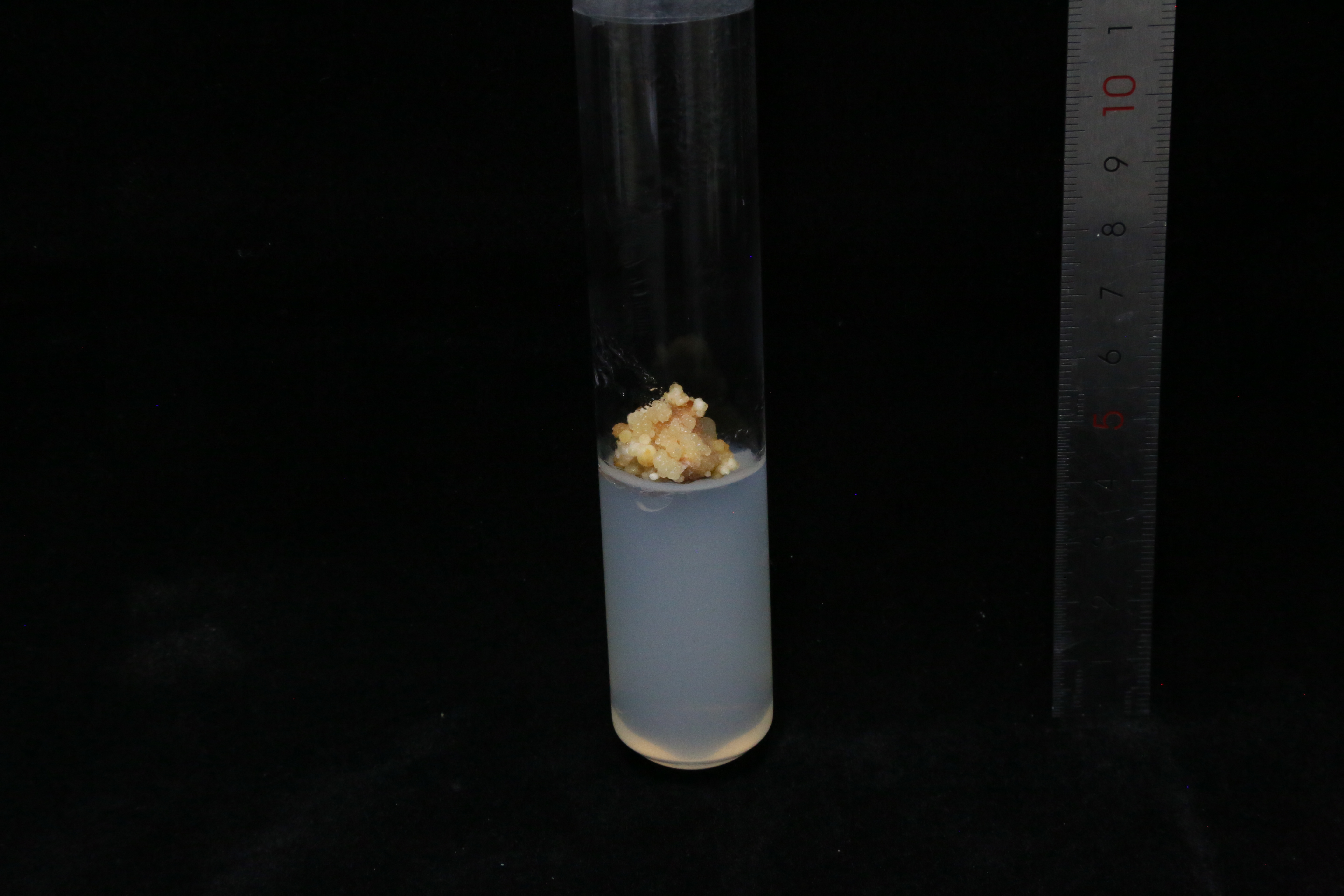


**Fig.S2** **Callus was induced from apical bud-derived tissues.**


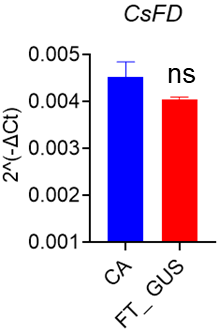


**Fig.S3 The expression levels of *CsFD* were assessed between non-transformed callus and callus overexpressing *CsFT3-like*.** CA, Callus used as the control. FT_GUS, Callus overexpressed *CsFT3-like*.


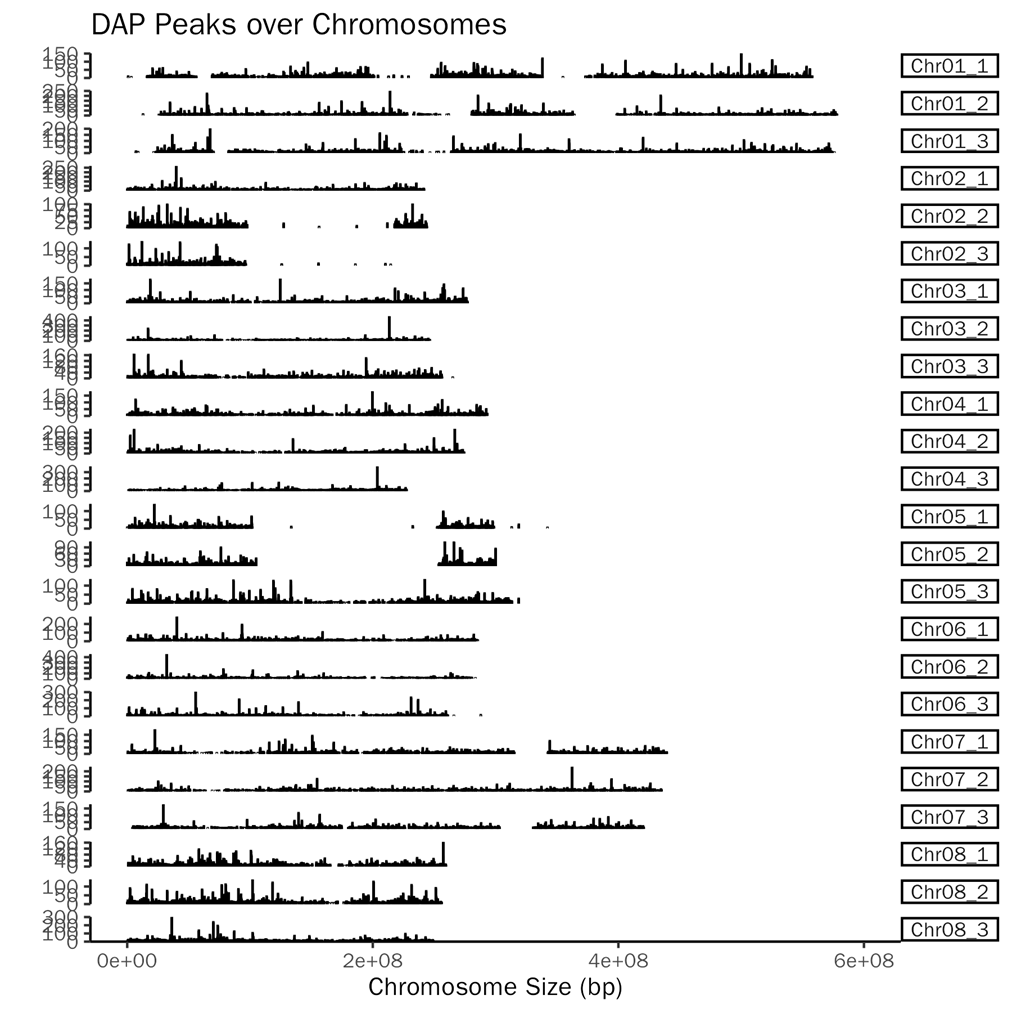


**Fig.S4** **Distribution map of identified peaks in different saffron chromosomes.**


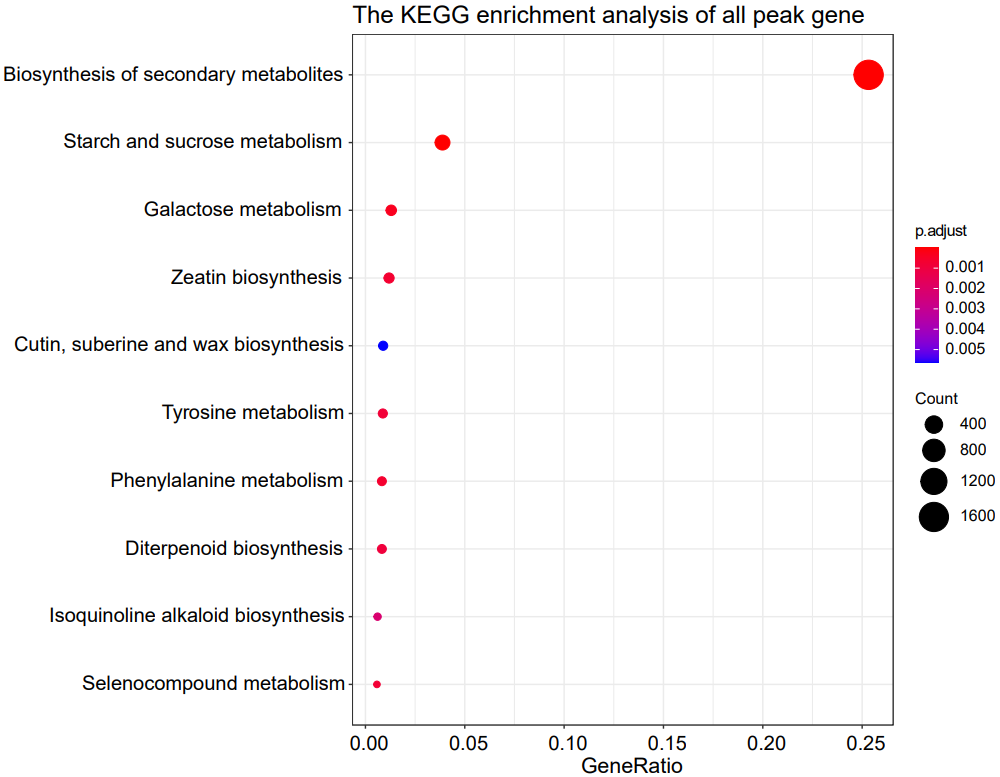


**Fig.S5 KEGG pathway enrichment analysis of candidate target genes of CsFT3-like-FD complex.** The color of the dots indicates the p-value, while the size of the dots corresponds to the number of genes.


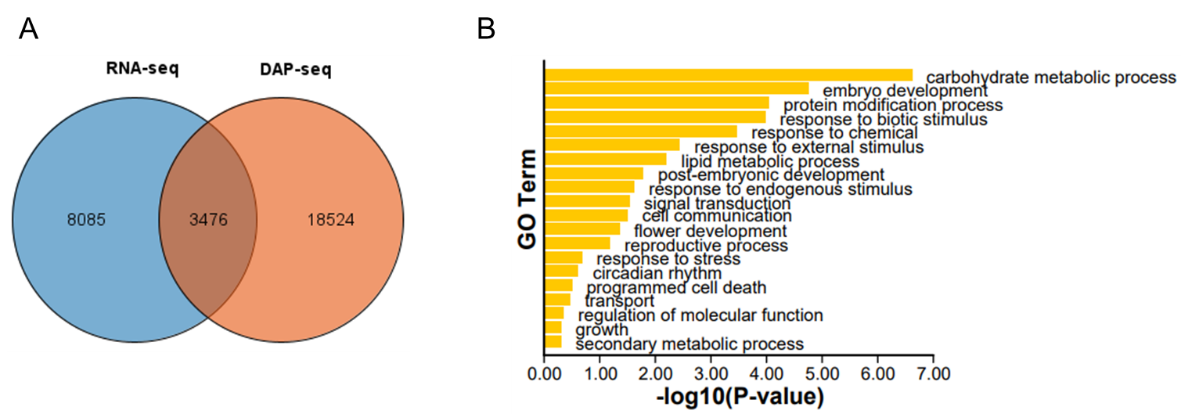
**Fig.S6 Identification of the targets** **of** **CsFT3-like-FD complex using RNA-seq and DAP-seq.** (A) Venn diagram of the CsFT3-like-FD complex target genes in DAP-seq analysis and DEGs between CA (Callus used as the control) and FT_GUS (Callus overexpressed *CsFT3-like*) in RNA-seq analysis. DEGs showing at least a 2-fold upregulation were defined as those with a |log₂ fold change| of ≥1.0 and p < 0.05. (B) The top20 enriched GO biological categories of target genes of CsFT3-like-FD complex.

**Fig.S**
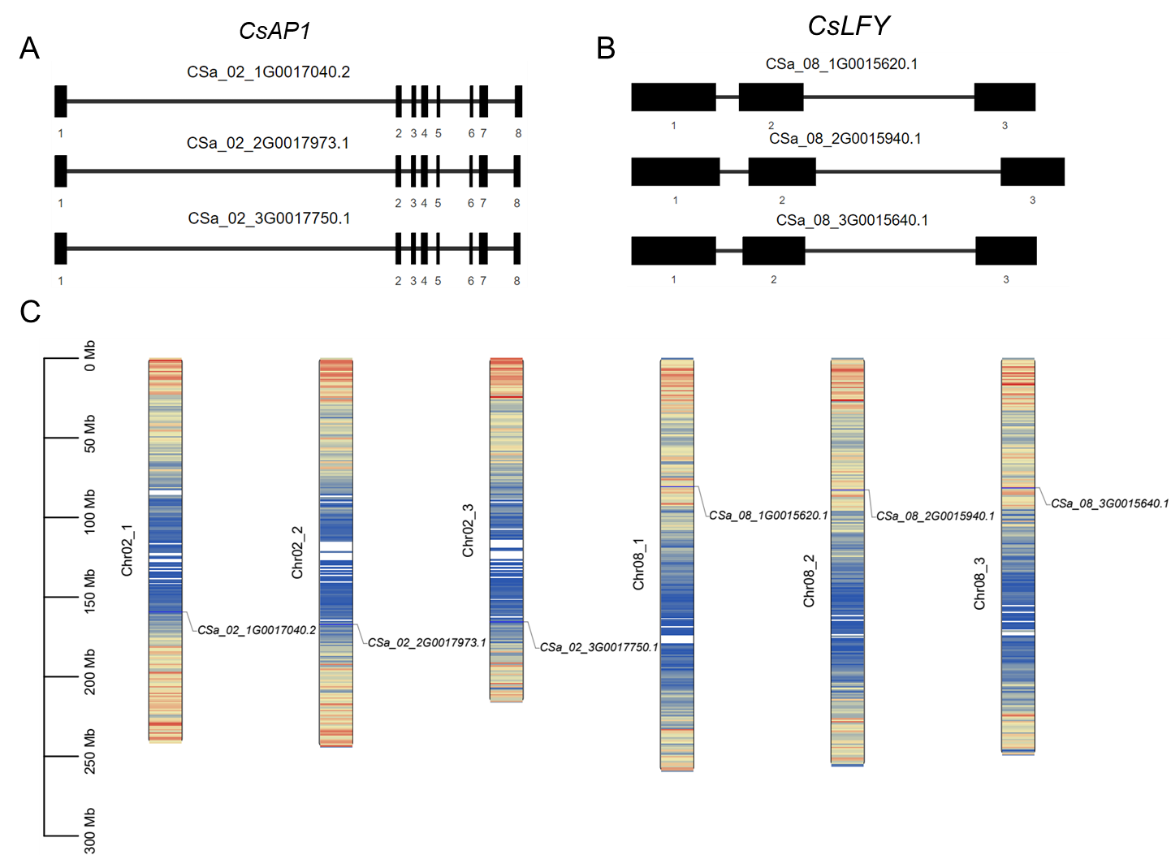
**7 The gene structures and chromosomal location distributions of *CsAP1* and *CsLFY* genes.** The gene structures of *CsAP1* (A) and *CsLFY* (B). They have three alleles. Black boxes represent exons, and black lines represent introns. (C) Distributions and localizations of *CsAP1* and *CsLFY* across chromosomes. The left scale represents chromosome length.


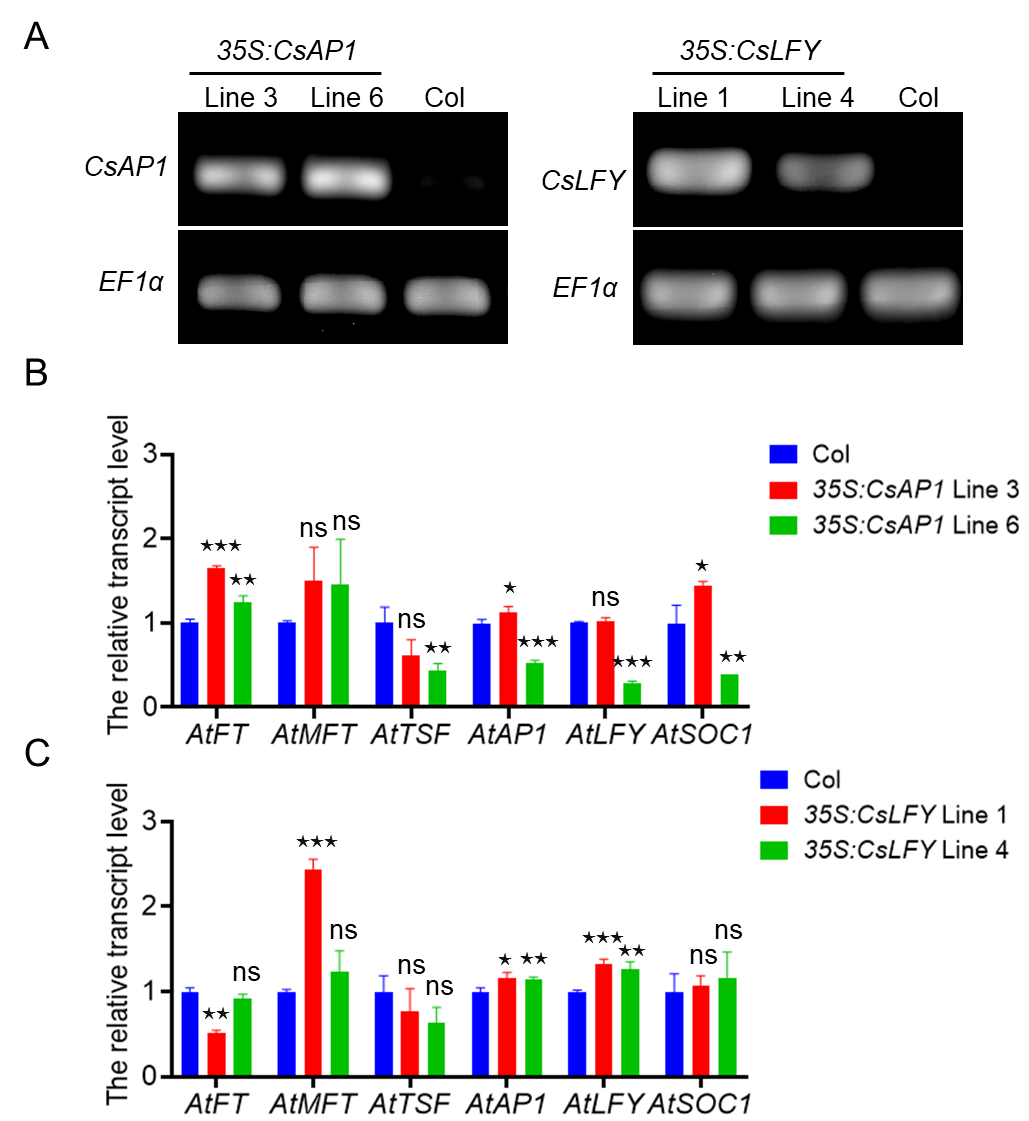


**Fig.S8** **Relative gene expression of endogenous flowering-related genes.** (A) Semiquantitative RT-PCR analysis of wild-type, and *CsAP1* and *CsLFY* transgenic *Arabidopsis thaliana*. Expression of the flowering related genes in inflorescence of *Arabidopsis* wild-type, *35S:CsAP1* (B) and *35S:CsLFY* (C) transgenic lines. The data are represented as the means ± SD, with three replicates (n = 3). Statistical significance was assessed using the Student’s t-test. **P* < 0.05, ***P* < 0.01, ****P* < 0.001, ns, no significance.


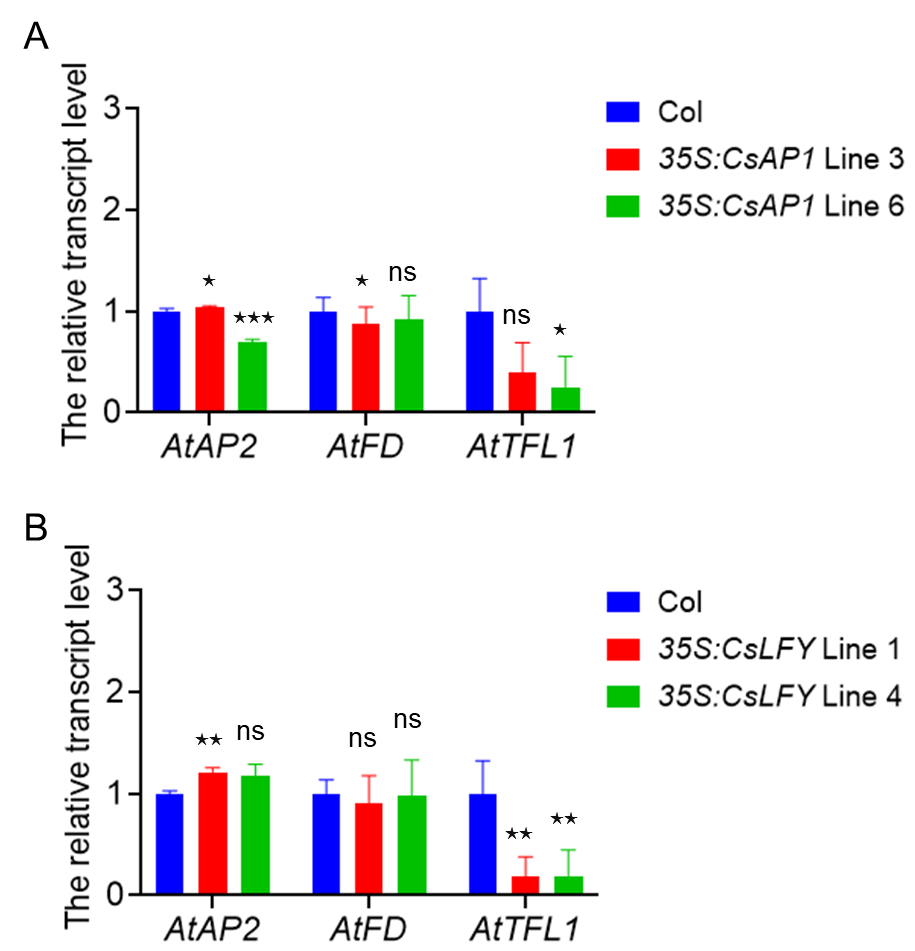


**Fig.S9** **Relative gene expression of endogenous flowering-related genes.** Expression of the flowering related genes *AtAP2*, *AtFD* and *AtTFL1* in inflorescence of *Arabidopsis* wild-type, *35S:CsAP1* (B) and *35S:CsLFY* (C) transgenic lines. The data are represented as the means ± SD, with three replicates (n = 3). Statistical significance was assessed using the Student’s t-test. **P* < 0.05, ***P* < 0.01, ****P* < 0.001, ns, no significance.

**
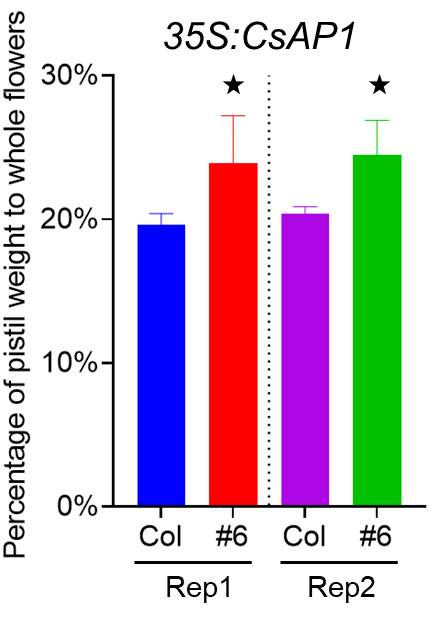
**

**Fig.S10 Changes in the pistil-to-total floral weight ratio were observed in *Arabidopsis* wild-type and *35S:CsAP1* transgenic plants.**

**Table S1 The percentage of plants exhibiting floral organ developmental defects.**

| Category | Number of plants | Percentage |
| --- | --- | --- |
| Floral organs with developmental defects | 60 | 67% (60/89) |
| Petals absent, stamens and pistils present, sepals 0-2 | 18 | 30% (18/60) |
| increased petals, stamens and pistils | 37 | 62% (37/60) |
| More Stamens, No Petals/Sepals | 37 | 67% (60/92) |

**Table S2 Primers used in this study**

| **Name** | **Primer (5’-3’)** | **Application** |
| --- | --- | --- |
| mGFP 172 R | CAGGTAGTTTTCCAGTAGTGCAAATA | Genotyping |
| *35S:CsFD* F | GCTTTCGCGAGCTCGGTACCATGTGGTTCTCTTCGGTACAAGAG | Phenotypic analysis |
| *35S:CsFD* R | AGGTCGACTCTAGAGGATCCTCAAAATGGAGCTGTTAAGGTTCT | Phenotypic analysis |
| *35S:CsAP1* F | GCTTTCGCGAGCTCGGTACCATGGGAAGGGGTAGGGTACAG | Phenotypic analysis |
| *35S:CsAP1* R | AGGTCGACTCTAGAGGATCCCTATCCATTAACAGAGCGAATCATC | Phenotypic analysis |
| *35S:CsLFY* F | gagctttcgcgagctcggtaccATGGACGCAGGAGGAGGAG | Phenotypic analysis |
| *35S:CsLFY* R | CCTGCAGGTCGACTCTAGAGGATCCCTAGAAAATCGGTGGTGGC | Phenotypic analysis |
| CsFD-1302 F | ACACGGGGGACTCTTGACATGTGGTTCTCTTCGGTACAAGA | Subcellular localization |
| CsFD-1302 R | cctttactagtcagatctacCATAAATGGAGCTGTTAAGGTTCTCT | Subcellular localization |
| CsAP1-1302 F | acacgggggactcttgacATGGGAAGGGGTAGGGTACA | Subcellular localization |
| CsAP1-1302 R | cctttactagtcagatctacCATTCCATTAACAGAGCGAATCA | Subcellular localization |
| CsLFY-1302 F | acacgggggactcttgacATGGACGCAGGAGGAGGAG | Subcellular localization |
| CsLFY-1302 R | CCTTTACTAGTCAGATCTACCATGAAAATCGGTGGTGGCA | Subcellular localization |
| *Tubulin* Q F | CGTGCGTTTGTTCACTGGTA | qPCR |
| *Tubulin* Q R | CCCACCTCTTCGTAATCCTTC | qPCR |
| *qCsFD* F | CCACCTGCACCTGCACTGAG | qPCR |
| *qCsFD* R | GCCATGGCTTGAGCTGTCG | qPCR |
| *qCsAP1* F | CGACGTGGCACTCATCATCTT | qPCR |
| *qCsAP1* R | CAGCCTCTGGTACTGCAATCG | qPCR |
| *qCsLFY* F | TCGACGAGGCCATTCTACTCT | qPCR |
| *qCsLFY* R | AGCTGCCTCGTCCTTCCTC | qPCR |
| *EF1α* Q F | AAATACTCCAAGGCTAGGTACG | qPCR |
| *EF1α* Q R | AAATGGGATTTTGTCAGGGTTG | qPCR |
| *AtFT* Q F | TCCTAGCAACCCTCACCTCC | qPCR |
| *AtFT* Q R | GCCACTCTCCCTCTGACAAT | qPCR |
| *AtMFT* Q F | CATCACTAACGGCTGCGAGA | qPCR |
| *AtMFT* Q R | CGGTGAGTTTTGCCGGAAAA | qPCR |
| *AtTSF* Q F | GATCCTCTTGTGGTCGGCAG | qPCR |
| *AtTSF* Q R | CATTTCCAGTGGTGGCAGGT | qPCR |
| *AtAP1* Q F | TGCTCTTGTTGTCTTCTCCCA | qPCR |
| *AtAP1* Q R | AAGAGCAGTGTCAAGCTGCT | qPCR |
| *AtLFY* Q F | TCAAAGCTGCCGTTAGAGCT | qPCR |
| *AtLFY* Q R | TTTCTCCGTCTCTGCTGCTG | qPCR |
| *AtSOC1* Q F | GCTCTCAGTGCTTTGTGATGC | qPCR |
| *AtSOC1* Q R | AATCTGTTGCAGCTCCTCGA | qPCR |
| AD-CsFD F | tggccatggaggccagtgGCATGTGGTTCTCTTCGGTAC | Y2H |
| AD-CsFD R | tgcagctcgagctcgatgTCAAAATGGAGCTGTTAAGGTTCT | Y2H |
| BD-CsFT3-like F | ggaggccgaattcccgggCATGGCTGCTGCTACTAGAGA | Y2H |
| BD-CsFT3-like R | agttatgcggccgctgcaTCAAGCGTACGTTCTCCTCC | Y2H |
| CsFD-2306 F | acgagctcggtacccgggATGTGGTTCTCTTCGGTACAAGA | Co-IP |
| CsFD-2306 R | gatccaagggcgaattggCCAAATGGAGCTGTTAAGGTTCT | Co-IP |
| CsFT3-like 1302 F | ACACGGGGGACTCTTGACATGGCTGCTGCTACTAGAGA | Co-IP |
| CsFT3-like 1302 R | CCTTTACTAGTCAGATCTACCATAGCGTACGTTCTCCTCCC | Co-IP |
| CsFT3-like 1303 F | ACACGGGGGACTCTTGACATGGCTGCTGCTACTAGAGA | GUS |
| CsFT3-like 1303 R | CGTAAACTAGTCAGATCTACCATAGCGTACGTTCTCCTCCC | GUS |
| *CsAP1pro*-0800 F | atagggcgaattgggtacTTGAGAGGCATTATATAAGAATGACCT | Dual-LUC |
| *CsAP1pro*-0800 R | gccgctctagaactagtgCGATCGAATCGCCCAAAATAGA | Dual-LUC |
| *CsLFYpro*-0800 F | atagggcgaattgggtacCCCTAATATGATGTATATATAGAATTAGGGT | Dual-LUC |
| *CsLFYpro*-0800 R | gccgctctagaactagtgCTTCTTCTTGTCTTACTCTTCTCTAGC | Dual-LUC |
| 5941-CsFT3-like F | cattacaattacatttacaattacATGGCTGCTGCTACTAGAGA | Dual-LUC |
| 5941-CsFT3-like R | ctctctagactcacctagTCAAGCGTACGTTCTCCTCC | Dual-LUC |
| 5941-CsFD F | cattacaattacatttacaattacATGTGGTTCTCTTCGGTACAAGA | Dual-LUC |
| 5941-CsFD R | ctctctagactcacctagTCAAAATGGAGCTGTTAAGGTTCT | Dual-LUC |
